# In Situ Derived Impedance–Structure Correlation during LaNiO_3_ Decomposition

**DOI:** 10.1021/jacs.5c16809

**Published:** 2025-11-20

**Authors:** Christoph Malleier, Thomas F. Winterstein, Marc Heggen, Volker Kahlenberg, Bernhard Klötzer, Simon Penner

**Affiliations:** † Institute of Physical Chemistry, University of Innsbruck, Innrain 52c, A-6020 Innsbruck, Austria; ‡ Ernst Ruska-Centre for Microscopy and Spectroscopy with Electrons, 28334 Forschungszentrum Jülich GmbH, Leo-Brandt-Str. 1, D-52428 Jülich, Germany; § Institute of Mineralogy and Petrography, University of Innsbruck, Innrain 52d, A-6020 Innsbruck, Austria

## Abstract

We demonstrate that in situ impedance spectroscopy is a marker method to follow LaNiO_3_ decomposition upon hydrogen reduction and is highly potent for the in situ detection of bulk- and surface-located chemical and structural transitions. Combined with in situ X-ray diffraction (XRD), it simultaneously proved the possibility to assess the electrochemical properties of oxygen-deficient phases and the full decomposition products La_2_O_3_ and Ni. In situ correlation of impedance and differential thermoanalytic data allows quantitatively pinpointing distinct exothermic peaks to LaNiO_2.5_ and La_2_O_3_ + Ni formation. The initial impedance increase at low temperatures is related by in situ near-ambient pressure X-ray photoelectron spectroscopy to near-surface redox transformations. Equilibrium impedance investigations revealed a pronounced kinetic delay in the structural transformations at low temperatures. In situ impedance spectroscopy upon redox cycling between reductive (H_2_) and oxidative (O_2_) conditions allowed us to clearly discriminate between reversible and irreversible transformations and demonstrated exceptional sensitivity to surface reorganization, including the reoccupation of oxygen vacancies and recompensation of structural defects. Frequency-dependent investigations demonstrate that LaNiO_3_ exhibits an inductive reactance in O_2_. Formation of oxygen-deficient LaNiO_2.5_ and irreversible decomposition into La_2_O_3_ + Ni are reflected in the frequency-dependent investigations and expressed via increasing capacitance. p-type semiconduction profoundly influences the impedance behavior of NiO in oxidative and reductive atmospheres and was found to be the key conduction contribution of LaNiO_3_ decomposition at 600 °C. Our work highlights the strength of in situ impedance spectroscopy as a noninvasive, highly responsive marker for surface chemistry, defect dynamics, and bulk structural transformations during redox experiments in perovskites, as evidenced for LaNiO_3_.

## Graphical abstract

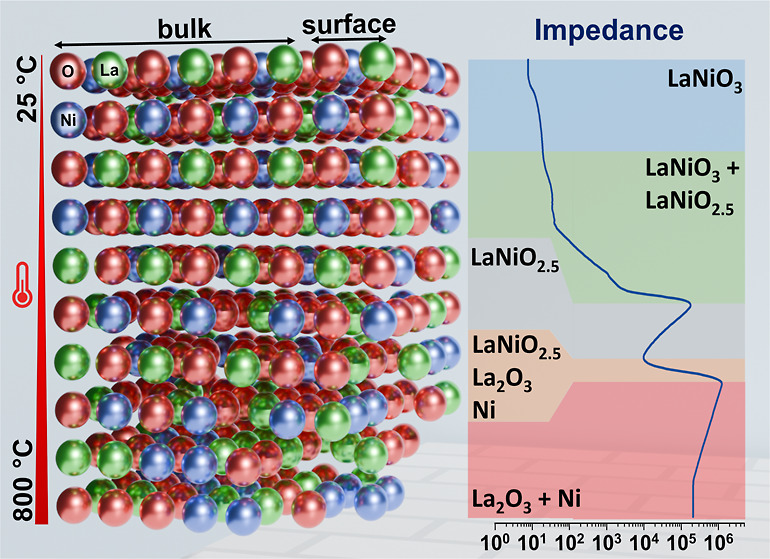

## 1 Introduction

In recent years, there has been growing interest in utilizing precursor structures to synthesize catalytically active and selective materials through deliberate and controlled decomposition under relevant catalytic reaction environments.^1–3^ A range of catalytic materials, encompassing the corrosion and self-activation of intermetallic compounds or the decomposition of perovskite frameworks, has been extensively studied in a variety of catalytic processes.^4,5^ A distinguishing characteristic of catalytic perovskite materials is the potential gas-phase-dependent instability of their structure. Often regarded as an undesirable feature, it has been demonstrated that it can be strategically exploited to modify metal–oxide interfaces, provided the perovskite decomposition is executed under controlled conditions.^4,6^ The perovskite structure is thus considered a precursor, whose breakdown is governed by the generation of the catalytically active phase. Typically, such a preparation strategy results in a metal–oxide system characterized by a defined interface, assuming complete decomposition of the perovskite, or by a metal–perovskite interface in the case of partial decomposition. In either scenario, the resultant catalytic material exhibits a significantly more complex structure and morphology.

A critical aspect of this decomposition process involves the exsolution of metal nanoparticles from the perovskite lattice as an initial step followed by the eventual collapse of the perovskite framework.^6^ Preceding or accompanying nanoparticle exsolution, polymorphic transitions, formation of oxygen-deficient structures, or transient phases are also observed.^4^ It is essential to note that these structural modifications play a pivotal role in the formation of the final active phase, as documented for a series of (doped) La–Ni perovskite materials.^2,7^ For LaNiO_3_, structures during decomposition include specific oxygen-deficient phases, such as LaNiO_2.7_, LaNiO_2.5_, or La_2_NiO_4_, which represents an important/crucial intermediate structure prior to the complete collapse of the perovskite structure.^8^

A variety of different in situ methods are at hand to monitor the structures that form during perovskite decomposition including classical structure-determining methods such as in situ X-ray diffraction and electron microscopy. Drawbacks of these methods are contrast issues which make the identification of simultaneously present phases difficult or the essential limitation to the characterization of crystalline phases. Especially for LaNiO_3_, this limitation can be overcome by employing a method that is sensitive to the specific physicochemical characteristics of LaNiO_3_ and its decomposition products. LaNiO_3_ exhibits the unique electronic property being the only rare earth nickelate to stay metallic and paramagnetic down to the lowest temperatures.^9–11^ Apart from LaNiO_3_, all related RNiO_3_ (R = Pr–Lu, Y) materials feature a metal–insulator transition at lower temperatures with an insulating antiferromagnetic ground state. With La exhibiting the largest ionic radius of the series, it was predicted to feature the strongest antiferromagnetic properties, as decreasing Ni–O–Ni bond angles alter the electronic bandwidth and magnetic exchange interactions. However, LaNiO_3_ entirely violates the general trend and does not show any magnetic ordering upon cooling, therefore remaining paramagnetic down to the lowest temperatures with an enhanced effective mass.^9^

Due to these unique yet hardly understood electronic properties of LaNiO_3_ behaving very much like a conventional metallic conductor, we have used in situ electric impedance spectroscopy to monitor the impedance and conduction changes occurring during the catalytically relevant perovskite decomposition and formation of transient structures with different electronic conduction properties in the present contribution. We show that during reduction in hydrogen, the changes in the electronic conduction properties are prominent along the decomposition pathway, so that impedance spectroscopy can effectively be used as a marker method to much more accurately pinpoint structural transformations and both the presence of specific oxygen-deficient phases and its decomposition products La_2_O_3_ and Ni than classical structure-determining methods. In addition, by relating the impedance changes to structural and differential thermoanalytic data derived from in situ X-ray diffraction (XRD), in situ X-ray photoelectron spectroscopy (XPS), and in situ differential thermo-analysis (DTA), we are able to establish reliable impedance–structure relationships.

## 2 Experimental Section

### 2.1 Synthesis of Materials

LaNiO_3_ was obtained by a modified Pechini method^12^ using a metal-ion to citric acid ratio of 1:2 and a metal-ion to ethylene glycol ratio = 1:4. For the synthesis, La­(NO_3_)_3_·6H_2_O (Carl Roth, 99.99%) and Ni­(NO_3_)_2_·6H_2_O (Puratronic, 99.9985%) were dissolved in water and citric acid. The lanthanum–nickel solution was heated and continuously stirred at 60 °C until all products dissolved. The mixture was further heated at 120 to 140 °C under permanent stirring until a green–dark-brown gel formed. The gel was then calcined in air at 450 °C for 4 h. The resulting ashes were ground to a powder in an agate mortar and sintered at 800 °C for 17 h. After sintering, the sample was ground up again, resulting in a black homogeneous powder. The usual stoichiometry of rhombohedral LaNiO_3_ is described as slightly oxygen-deficient and has been determined in previous works to range between LaNiO_2.94_ and LaNiO_2.88_.^13,14^ For the sake of clarity, we will use “LaNiO_3_” throughout the manuscript.

### 2.2 In Situ Electric Impedance Spectroscopy (EIS)

The in situ impedance cell consists of an outer quartz tube with two inner quartz tubes, to which the sample and the electrodes are attached to. Heating is provided by a tubular Linn furnace and controlled by a thermocouple (K-element), located in the reactor about 5 mm downstream of the sample, and a Linn High Therm 800P temperature controller. For impedance measurements, a Zennium XC impedance spectrometer (Zahner) is used, providing data on the impedance and the phase angle of the current as a function of voltage. The pressure-pelletized powder samples (pressure 125 MPa, 5 mm diameter, 1 mm thick, and sample mass 40 mg) are placed between two circular Pt electrodes. These form a plate capacitor in mechanically enforced contact with the sample pellet. For all temperature-programmed impedance measurements, an amplitude of 20 mV of the superimposed sinusoidal modulation voltage signal at an overall DC potential of 0 V and a frequency of 1 Hz is applied to the Pt electrodes. The impedance of the pellet is thus effectively measured in an electrochemically unpolarized state. In all temperature-dependent experiments, the impedance modulus value |*Z*| will therefore be further referred to as “impedance”. Arrhenius analysis was performed to determine the activation energies for conduction (*E*_A_s) for selected temperature regions. The conductivity was calculated from the reciprocal of the impedance modulus value multiplied with the sample length and divided by the sample’s cross-section area and subsequently plotted as ln­(conductivity) vs the reciprocal of the reaction temperature. This conductivity is generally proportional to the sum of the total charge carrier concentration and is not necessarily specific for a certain kind of charge carrier species. Hence, in the more frequent cases of mixed charge carrier conductance, only an “apparent” activation energy can be determined. As reported in this work, the calculated activation energy is usually a weighted sum of several contributions to the conductivity. Hence, there are generally too many parameters to unambiguously extract the individual activation energies of a single process, as several activated processes may occur simultaneously with unknown relative contributions. It may be possible only in exceptional cases to refer one specific activation energy to a single charge transport process. Thus, our focus is identifying qualitative changes in the activation energy that can eventually be clearly related to changes in the surface chemistry. A general remark on the temperature-dependent impedance behavior shown in the subsequent figures should be given at this point. As the detection limit of the used EI spectrometer is in the *G*Ω range, this especially limits the measurement of the semiconductive behavior in the low-to-medium temperature regions. The usual appearance is then a quasi-constant impedance behavior at the instrumental limit as a function of temperature before the impedance decreases due to the already ongoing increase of the thermally excited charge carrier concentration. It is thus necessary to state that the actual threshold temperature for activation of these charge carriers might be overcome at even lower temperatures, which is out of the detection limit of the spectrometer. Gases were supplied by Messer (He 5.0, O_2_ 4.5, H_2_ 5.0). A flow of 10% H_2_ or O_2_ in He under ambient conditions, controlled through MKS mass flow controllers, was used.

### 2.3 In Situ X-ray Diffraction (XRD)

In situ analysis of LaNiO_3_ in a flow of 10% H_2_ or O_2_ in He under ambient conditions, controlled through MKS mass flow controllers, up to 800 °C was carried out using a Rigaku SmartLab instrument (theta/theta geometry, Cu Kα_1,2_) using a HyPix-3000 detector in 1D scan mode with a Reactor X high temperature attachment under the exact same conditions as the impedance measurements with respect to gas flow, heating ramp, and gas composition. The powder was placed in a quartz sample holder with continuous patterns being recorded in parallel beam alignment in a range of 15° to 70° 2θ with a step width of 0.01°. For quantitative analysis, the program TOPAS 7.0 by Bruker was utilized to analyze the X-ray diffraction patterns using Rietveld refinement with a full axial model.^15^ A Double-Voigt approach was applied to calculate the crystallite sizes. The resolution function of the diffractometers was obtained from the structural refinement of a LaB_6_ standard.

### 2.4 In Situ Near-Ambient Pressure X-ray Photoelectron Spectroscopy (NAP-XPS)

In situ surface chemical analysis was carried out in a customized UHV system for in situ XPS applications (SPECS GmbH). The measurement chamber is composed of a μFOCUS 600 monochromatic small spot (100 × 300 μm^2^) Al K_α_ X-ray source, a hemispherical energy analyzer (PHOBIOS 150 NAP) in a vertical configuration, and a μ-metal analyzing chamber, shielding the system from external magnetic fields. To investigate polycrystalline samples, a pressed pellet covering a stainless-steel grid as a stabilizer is fixed on a sample-holder by mounting the pellet via a front plate. The excited photoelectrons were collected through a 300 μm nozzle directly from the sample’s frontside surface, which is fixed by a front plate with an 8 mm opening to the sample plate. Details of the apparatus are given in ref 16; qualitative analysis was based on the Ni 2p, La 3d, O 1s, La 4d, Ni 3s, and Ni 3p high-resolution spectra. Chemical shifts were calibrated to the signal of adventitious carbon component at 284.5 eV. Fitting of the Ni 2p, La 3d, O 1s, La 4d, Ni 3s, and Ni 3p spectra by different components and oxidation states was performed using literature-reported constraints for the full-width at half-maximum and the binding energies. Background correction was done using Shirley-type functions. Pure hydrogen at 100 Pa adjusted through Bronkhorst HighTech mass flow controllers was used.

### 2.5 Scanning Transmission Electron Microscopy (STEM)

Scanning transmission electron microscopy (STEM) characterization via high-angle annular dark field (HAADF) imaging and energy-dispersive X-ray analysis was carried out on a C_s_-aberration corrected (Ceos DCOR) FEI Titan G2 80-200 ChemiSTEM electron microscope operated at 200 kV at the Ernst Ruska-Centre Jülich employing an in-column Super-X energy-dispersive X-ray spectroscopy (EDX) unit (ChemiSTEM technology).

### 2.6 In Situ Differential Thermal Analysis (DTA)

(Derivative) differential thermal analysis ((D)­DTA) was carried out using a vacuum-tight NETZSCH STA Jupiter 449 F1 apparatus using a top-loading balancing geometry. The samples are placed in a corundum sample holder inside a SiC furnace (heating up to 1600 °C possible, S-type thermocouple control), which is both thermally isolated from the environment as well as sealed off from the atmosphere. The effectiveness of the seal has been confirmed previously by attaching a mass spectrometer and measuring the exhaust gases. Gases are admitted through three integrated mass-flow controllers. The temperature resolution of the apparatus is 0.001 K, the resolution of the balance 0.025 μg. The drift of the balance is <2 μg h^–1^. For in situ analysis during hydrogen reduction, measurements were conducted from 50 °C to 800 °C at 10 °C min^–1^ in a flow of 10% H_2_ in Ar under ambient conditions monitored through MKS mass flow controllers.

## 3 Results and Discussion

### 3.1 Impedance–Structure Relationships during Reductive LaNiO_3_ Transformation Derived from Combined In Situ Electric Impedance Spectroscopy and In Situ X-ray Diffraction

To investigate the impedance response to reduction-induced structural transformations of LaNiO_3_ and the associated dynamic exsolution of Ni, Figure 1 shows the in situ monitored electric impedance profile (top left) in direct correlation with the in situ collected PXRD patterns, highlighted as a 2D contour plot (bottom left). Before introduction of hydrogen, LaNiO_3_ shows the anticipated metallic conductivity behavior at 25 °C under a helium atmosphere. After switching to hydrogen, a slight increase in impedance is observed. After equilibration at 25 °C, LaNiO_3_ was heated to 800 °C, and an isothermal period of 30 min followed. During heating, the impedance shows a characteristic profile corresponding to distinct surface and bulk structural transformations, which give rise to associated changes in the impedance. Initially, LaNiO_3_ exhibits metallic conductivity also in hydrogen (under both He and O_2_ atmospheres up to 800 °C, metallic conductivity is maintained, Figure S2). To correlate the impedance changes to surface- and bulk-structural changes, complementary in situ PXRD and in situ XPS (cf. Figure 3) measurements were performed. Overall, the impedance profile can be separated into five different regions (shaded green, yellow, blue, light gray, and red), and thin characteristic changes in the impedance are detected, as marked by the letters A–G. Between 25 and 200 °C, the impedance steadily rises, followed by a steep increase by almost 5 orders of magnitude through points B (300 °C), C (roughly 350 °C), and D (400 °C) with semiconducting behavior. Between points D and E (500 °C), the impedance drops by 1 order of magnitude and rises again by 2 orders of magnitude to point F (570 °C). At higher temperatures, it drops again to point G (800 °C) and remains constant in the isothermal period at 800 °C. In due course, we are able to correlate each of these impedance changes to associated changes in the chemical state of the surface and the bulk structure. As for the latter, the data are shown as a two-dimensional contour intensity plot in the lower left panel of Figure 1, with direct temperature match to the impedance profile. In short, all impedance changes can be correlated to structural changes observed during the decomposition of LaNiO_3_. Selected X-ray diffractograms at distinct points A–G are shown in the right panel. Diffractograms A and B both feature pure LaNiO_3_.^17^ Note that we could not resolve the rhombohedral-to-cubic LaNiO_3_ phase transformation (between 230 and 250 °C) and the intermediate formation of LaNiO_2.7_ (between 250 and 280 °C) detected previously by the much higher resolved in situ synchrotron-based PXRD.^2^ Diffractogram C already indicates formation of LaNiO_2.5_.^18^ Diffractogram D suggests almost pure LaNiO_2.5_ with slight traces of remaining LaNiO_3_ and diffractogram E features the characteristic PXRD pattern for LaNiO_2.5_. Diffractogram F additionally shows characteristic reflections for La_2_O_3_(trig.),^19^ as well as metallic Ni.^20^ Diffractogram G shows more pronounced reflections for La_2_O_3_ and Ni but otherwise matches diffractogram F. We emphasize that the forking of the reflections between points B and D (very well visible at 47° and 33° 2θ) consists of two steps: first, still some reflections of LaNiO_3_ are visible until point C, and second, although the split into the reflections has already occurred, the phase transition is not yet fully completed. This characteristic forking is also visible in the impedance data, with two different slopes from B to D, separated by C at the exact temperature where the reflections of LaNiO_3_ dissipate. Overall, the data reveal a two-step decomposition pathway into La_2_O_3_ and metallic Ni:

$$LaNiO_{3}⇌LaNiO_{2.5}+0.5O$$

$$2LaNiO_{2.5}\to {La}_{2}O_{3}+2{Ni}^{0}+O_{2}$$

**Figure 1:**
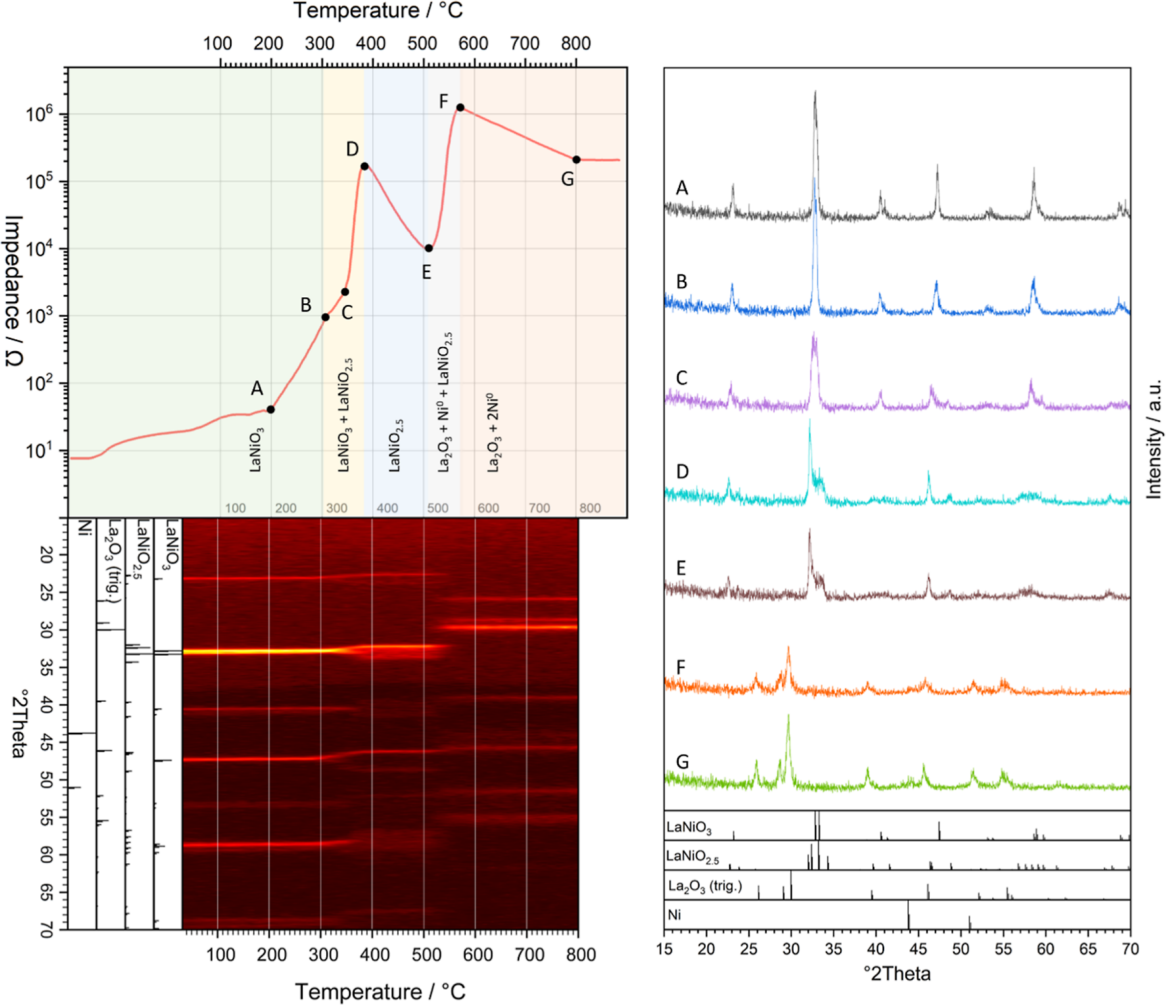
Top left: In situ impedance profile on LaNiO_3_ during reduction in 10% H_2_ in He under ambient conditions between 25 and 800 °C (heating rate 10 °C min^–1^) using 1 Hz perturbation frequency. Bottom left: Complementary in situ PXRD experiment between 15° and 70° 2θ conducted under the exact same reaction conditions displayed as a two-dimensional intensity plot to mark structural and phase transitions. The reaction axes of both experiments have been matched to visualize the impedance–structure correlation. In the right panel, representative PXRD patterns are shown at selected points of the impedance profile (points labeled as A–G). As a guide for the eye, dashed lines are shown in 100 °C steps for better correlation to the impedance data. The respective Rietveld refinement to qualitatively pinpoint the present phases is shown in Figure S1.

During the phase transformation processes, an increase in impedance is observed, in contrast to the decrease recorded during thermodynamically stable single-phase regimes. The impedance decrease is attributed to the intrinsic thermal activation of charge carriers characteristic of semiconducting materials, which exhibit enhanced conductivity upon heating.^20^ Conversely, the observed increase in impedance reflects the progressive evolution into structurally and electronically less conducting phases such as oxygen-deficient or multiphase intermediates. Under isothermal conditions (800 °C), the system reaches a steady state impedance, indicating the absence of further structural or electronic reorganization effects.

The quantitative decomposition of LaNiO_3_ at 380 °C and of LaNiO_2.5_ at 530 °C was further substantiated by in situ differential thermal analysis experiments. Figure 2 features the derivative differential thermal analysis (DDTA) data of LaNiO_3_ under similar in situ reduction conditions. The DDTA data corroborate the impedance data of Figure 1 by providing insight into the presence of two pronounced exothermal (labeled as points 1 and 2, respectively) and two endothermal signals (labeled as points 3 and 4, respectively) during the reduction process. By performing enthalpy calculations, the DDTA peak size relationship between these signals and structural transformations can be elaborated upon according to the following reaction network:^21–24^

**Figure 2:**
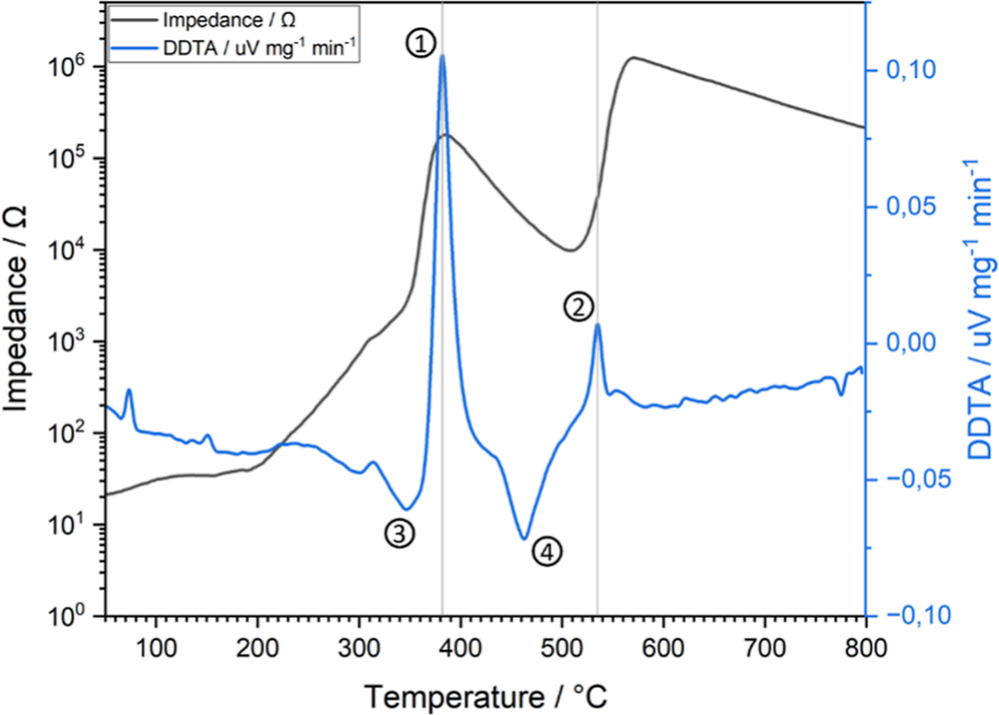
In situ impedance response of LaNiO_3_ in 10% H_2_ in Ar under ambient conditions from 50 °C to 800 °C superimposed with the complementary in situ DDTA profile obtained under identical reduction conditions. This comparative representation allows for direct correlation between thermochemical effects and electronic transport phenomena, highlighting the interplay between phase transitions and impedance behavior. The pronounced shoulder between points 3 and 4 is due to the overlapping processes of exothermic LaNiO_2.5_ formation and the endothermic Ni exsolution.

Point 1:

$${LaNiO}_{3}\left(s\right)+0.5H_{2}\left(g\right)\to {LaNiO}_{2.5}\left(s\right)+{0.5H}_{2}O\left(g\right)$$

$${Ni}^{3+}\to {Ni}^{2+},{\Delta H}_{298}^{0}=5.05\;kJ\;{mol}^{-1}$$

$$H_{2}+0.5O_{2}\to H_{2}O{,\Delta H}_{298}^{0}=-241.82\;kJ{\;mol}^{-1}$$

$${\Delta H}_{298,tot}^{0}=5.05\;kJ\;{mol}^{-1}+0.5\cdot \left(-241.82\;kJ\;{mol}^{-1}\right)=-115.86\;kJ\;{mol}^{-1}$$

Point 2:

$${LaNiO}_{2.5}\left(s\right)+H_{2}\left(g\right)\to {0.5La}_{2}O_{3}\left(s\right)+Ni\left(s\right)+H_{2}O\left(g\right)$$

$${Ni}^{2+}\to {Ni}^{0},{\Delta H}_{298}^{0}=239.70\;kJ\;{mol}^{-1}$$

$$H_{2}+0.5O_{2}\to H_{2}O,{\Delta H}_{298}^{0}=-241.82\;kJ\;{mol}^{-1}$$

$${\Delta H}_{298,tot}^{0}=239.70\;kJ\;{mol}^{-1}+\left(-241.82\;kJ{\;mol}^{-1}\right)=-2.12\;kJ{\;mol}^{-1}$$

Points 3 and 4:

$${Ni}^{2+}\to {Ni}^{0}{,\Delta H}_{298}^{0}=239.70\;kJ\;{mol}^{-1}$$

During the sequential phase transitions of LaNiO_3_ → LaNiO_2.5_ and LaNiO_2.5_ → Ni + La_2_O_3_, the DDTA data exhibit two distinct exothermic signals of different magnitudes, exactly matching the onset temperatures of the impedance data. The initial transformation from Ni^3+^ to Ni^2+^ during the transformation of LaNiO_3_ → LaNiO_2.5_ is a highly exothermic process for which the driving force is the instability of Ni^3+^ under hydrogen (Figure 2, enthalpy calculation 1 and Supporting Information Section D). LaNiO_2.5_, however, although releasing twice the amount of oxygen in the reduction to Ni^0^ + La_2_O_3_, is strongly extenuated by the enthalpically less favorable Ni^0^ state over thermodynamically stable Ni^2+^. This reduces the overall exothermicity. Nevertheless, a modest surplus in exothermic process enthalpy eventually remains, which is captured in the in situ DDTA experimental data, corroborating the impedance-derived transition temperature. Additionally, two endothermic events are also present in DDTA, prior to phase decomposition. These features mark the tipping points in chemical potential, where the exsolution of Ni^2+^ from the perovskite lattice starts to take place. Ni^2+^ species of the perovskite start to exsolve onto the surface, nucleate, and agglomerate into metallic Ni nanoparticles, while the perovskite lattice remains structurally intact but enriched in defect sites.

The endothermicity also corresponds to the classical nucleation theory which expounds that the nucleation enthalpy, in this case for metallic Ni, runs through a maximum in the energy barrier during the formation of a critical nucleus. Therein the critical nucleus size is a function of the energy gained by forming a stable phase in (scaling with *r* ^3^) and unfavorable surface energy needed to create a new surface (scaling with *r* ^2^). As the reduction into Ni^0^ in a H_2_ atmosphere is solely slightly exothermic, the nucleation enthalpy also contributes to the endothermicity recorded in the data.^25–27^

Although the associated reduction of Ni^2+^ to Ni^0^ is strongly endothermic, its appearance in the DDTA data is comparatively small, which the number of participating ions explains. The Ni exsolution process at points 3 and 4 is tightly linked to the surface, in contrast to the following phase transitions, which affect the entire bulk structure, leading approximately to the same magnitude in signal as the second bulk structural change. This interpretation is further substantiated by the in situ XPS measurements discussed in Section 3.2. The DDTA profile exhibits a number of reproducible small exothermic and endothermic signals, which can be explained by the removal of adsorbed species (below ca. 200 °C, e.g., water) and bulk structural transformations (rhombohedral into cubic LaNiO_3_ at 230 °C and transient formation of LaNiO_2.7_ between 250 and 280 °C).^2^

### 3.2 In Situ X-ray Photoelectron Spectroscopy as a Marker for Reduction-Induced Surface Alterations in LaNiO_3_

As impedance measurements have proven to be a sensitive marker for the structural deconvolution of LaNiO_3_ into LaNiO_2.5_ and Ni + La_2_O_3_, the question remains what processes underly the impedance increase between 25 and 280 °C (up to point B in Figure 1), where the PXRD data reveal phase-pure LaNiO_3_. In this context, the role of surface chemistry becomes pivotal to understanding early stage impedance evolution at low reduction temperatures. To further probe these surface transformations with element- and oxidation-state-specific resolution, in situ near-ambient pressure X-ray photoelectron spectroscopy (NAP-XPS) was employed, offering a direct investigation of the evolving surface chemistry and electronic structure during reduction. Although full (bulk) LaNiO_3_ decomposition is delayed due to the reduced pressure and not expected, it allows us to pinpoint the initial stages of reduction temporarily and, therefore, spectroscopically more easily.

The surface chemical and electronic properties were investigated by near-ambient pressure X-ray photoelectron spectroscopy at 100 Pa H_2_ pressure between 25 and 400 °C. The spectra are shown in Figure 3, and Table S2 is a full list of binding energies and fit parameters. The splitting of La 3d_3/2_ and Ni 2p_3/2_, overlapping at 25 and 100 °C, into two signals from 280 °C, stands out in the La 3d/Ni 2p region. A binding energy of 854.6 eV confirms the presence of Ni^3+^,^29^ which is expected in LaNiO_3_. The shift toward 852.7 eV binding energy confirms the occurrence of the exsolution process, as it shows the emergence of metallic Ni, evidenced by the appearance of a distinct third peak. The presence of metallic Ni on the surface can be solely attributed to the exsolution phenomenon, which markedly influences the electronic structure of LaNiO_3_. This surface-segregated Ni mimics a corrosion-like interfacial layer, analogous to degradation phenomena observed in electrode materials, and introduces a transition- or contact-related charge transfer resistance. The intensity of the metallic Ni component progressively increases up to 350 °C relative to the La 3d spectral features and remains stable through 400 °C, indicating a thermally sustained exsolution state. Further evidence of surface structural modification is provided by the O 1s region, which reveals a distinct separation into lattice oxygen, 529.6 eV, vacancy/defect oxygen, 531.1 eV, and surface oxygen, 532.7 eV,^29,31^ underscoring the evolving oxygen coordination environment and surface reconstruction during activation. Surface oxygen is a consequence of lower coordination to cationic sites and hydroxylation and is well-established at 25 °C. At 100 °C, surface alterations start to be already visible (indicated by the intensity gap in the O 1s signal), which we could, moreover, associate with surface and bulk oxygen deficiency contributing to the impedance increase (Figure 1), as LaNiO_3_ is slightly nonstoichiometric and intermediary also forms bulk LaNiO_2.7_ at temperatures between 230 and 280 °C.^2^ After the exsolution at 280 °C, almost all undercoordinated surface oxygen species are removed and culminate in the total removal of surface oxygen at 350 °C. Simultaneously, the lattice component, as well as the vacancy species, remains intact and hardly loses intensity, proving the stability of the remaining perovskite, in accordance with PXRD. Complementary insights are obtained from the Ni 3p spectral region, which further corroborates the exsolution of nickel: a distinct chemical shift from oxidized Ni^3+^ at 68.5 eV to metallic Ni^0^ at 66.4 eV provides compelling evidence for the reduction and emergence of metallic Ni, in alignment with the transformations observed in the Ni 2p region.^28–32^

**Figure 3:**
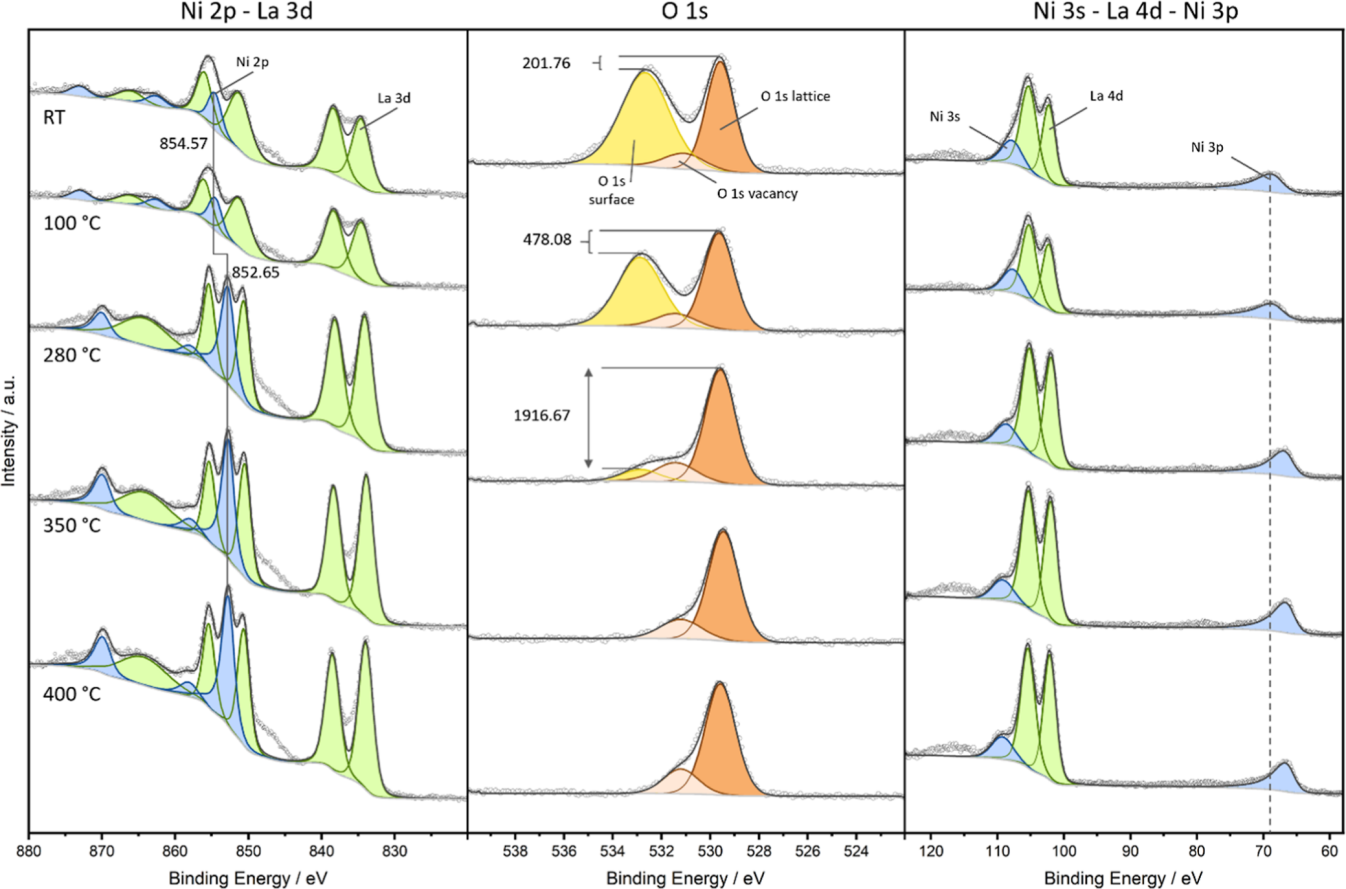
In situ high-resolution X-ray photoelectron spectra of the La 3d/Ni 2p, O 1s, and Ni 2s/La 4d/Ni 3p region for LaNiO_3_ in 100 Pa H_2_ from 25 to 400 °C. Spectra have been fitted with the corresponding components, peak shifts of Ni^2+^ to Ni^0^ indicated by the corresponding BE values and guidance lines.^28–30^ The diminishment of the surface O 1s component is emphasized by the intensity gap (in units cps).

### 3.3 Equilibrium-Driven In Situ Impedance Spectroscopy: Probing Structural Stability, Gas Phase Dependence, and Reactive Reversibility

Building upon the observations from the temperature-dependent impedance measurement (Figure 1), an equilibrium-based impedance spectroscopy analysis was conducted to further elucidate the thermodynamic and structural dynamics of LaNiO_3_ under redox conditions. Specifically, selected temperatures were extracted from the temperature-dependent investigations between 200 and 800 °C, corresponding to distinct structurally and chemically pertinent points:

- 200 °C: the bulk perovskite structure remains intact, as confirmed by PXRD (Figure 1), while surface-level modifications are already evident (see XPS, Figure 3).
- 400 °C: represents the completion of the phase transformation to LaNiO_2.5_.
- 600 °C: marks the near-complete decomposition into La_2_O_3_ and metallic Ni.
- 800 °C: serves as the final temperature of LaNiO_3_ in the temperature-dependent evaluation and simultaneously corresponds to the calcination temperature of LaNiO_3_.

In the graphical representation, dark-red coloring of the background indicates heating steps, light-red, equilibrium steps, and blue, the exposure to 10% O_2_ in He. The measurement procedure included the following steps (all under ambient conditions):

1. Heating the sample in 10% H_2_ in He to the target temperature,
2. Equilibrating the sample under isothermal conditions,
3. Exposure to 10% O_2_ in He (with intermediate He purging),
4. Equilibration under oxidation conditions,
5. Subsequent exposure to 10% H_2_ in He (with intermediate He purging), and
6. Equilibration until system stability is regained.

Profound changes are observed between the oxidation and reduction phases. At 200 °C, the impedance rises to 18 kΩ before and 13 kΩ after O_2_ exposure, which confirms the reversibility of this oxidation–reduction cycle. After the exposure to O_2_, the reduction back to around 10 kΩ proceeds more quickly than the initial reduction. Generally, equilibrium times are notably faster at higher temperatures with the significantly slowest equilibration time at 200 °C, underscoring the temperature sensitivity of the redox dynamics in LaNiO_3_.

Similar to the temperature-dependent measurement, the impedance runs through a maximum between 200 °C and 400 °C and equilibrates at 41 kΩ. The short peak before the exposure to O_2_ is a result of He purging. Under an O_2_ atmosphere, the impedance decreases to metallic behavior with 10 Ω back again, and after another H_2_ exposure, the impedance reversibly increases back, with a notable effect, the small maximum before equilibration being again indicative for the phase transition.

In the temperature range between 400 and 600 °C, the impedance response mirrors the results from the temperature-dependent evaluation. Initially, a decrease in impedance is found (attributed to the semiconductive nature of LaNiO_2.5_ during heating, specifically, the thermally activated charge carrier mobility increased conductivity), which is followed by a steep increase at 510 °C until it proceeds through another maximum at 590 °C and equilibrates at 600 °C at 1 MΩ. After the exposure to O_2_, the impedance decreases but only to 100 Ω, in contrast to ∼10 Ω seen in previous oxidation cycles. This is attributed to the formation of NiO, a well-characterized p-type semiconductor.^1,33,34^ Figure S2 provides additional measurements and discussion on NiO under inert (He), oxidation, and reduction conditions. A more in-depth look at this oxidation process at 600 °C between points E and F by in situ PXRD is given in Figure S3.

The temperature increase to 800 °C results in a decrease in impedance, consistent with the observations of the temperature-dependent measurement, and complies with the impedance characteristics of thermally enhanced semiconducting behavior. Upon exposure to O_2_, the reoxidation from La_2_O_3_ and Ni nanoparticles into the perovskite phases LaNiO_3_ and LaNiO_2.5_ and the nonstoichiometric Ni- and O-deficient La_4_Ni_3_O_10_ phase is initiated. A closer look at this transition is given in Figure S4, where the progression is monitored by in situ PXRD. Several distinct points, where the equilibrium states in H_2_ and O_2_ atmospheres of the samples were reached, are identified.

To again correlate impedance behavior and structural evolution, we have collected X-ray diffraction patterns at selected points along the reduction–oxidation axis (labeled as points A–H, Figure 4) to comprehensively correlate redox conditions, structural transitions, and electronic response (Figure 5). At points A and B, only LaNiO_3_ is present. Diffractogram C shows pure LaNiO_2.5_ in H_2_, which according to diffractogram D back-oxidizes fully to LaNiO_3_. Diffractogram E shows the characteristic reflections for La_2_O_3_(trig.),^19^ as well as metallic Ni, without any remaining LaNiO_2.5_ or LaNiO_3_. In diffractogram F, a drastic loss in overall intensity is observed. Furthermore, Ni was oxidized to NiO, as derived from the shift in PXRD 43.8° 2θ (Ni^0^)^18^ to 43.3° 2θ (NiO),^35^ and also mirrors the impedance value of the reference measurement on NiO (O_2_) in Figure S2 with 100 Ω. Diffractogram G features full back-transformation of La_2_O_3_ + NiO into La_2_O_3_ + Ni^0^, re-establishing diffractogram E and reregaining all features and intensity. This coherently proves the reversibility of the reaction step at 600 °C by switching back from an oxidizing atmosphere to a reducing atmosphere. By exposing the sample to oxygen at 800 °C (inset H), a different route of reaction is adopted compared to 600 °C: the La_2_O_3_ + Ni compositional mixture is not fully reoxidized. However, the temperature is high enough to partly readopt the perovskite formation reaction again, leading to a crystallization in the perovskite structure (LaNiO_3_) and yielding also the nonstoichiometric Ni- and O- deficient phase La_4_Ni_3_O_10_,^36^ NiO,^35^ and LaNiO_2.5_.^18^

**Figure 4:**
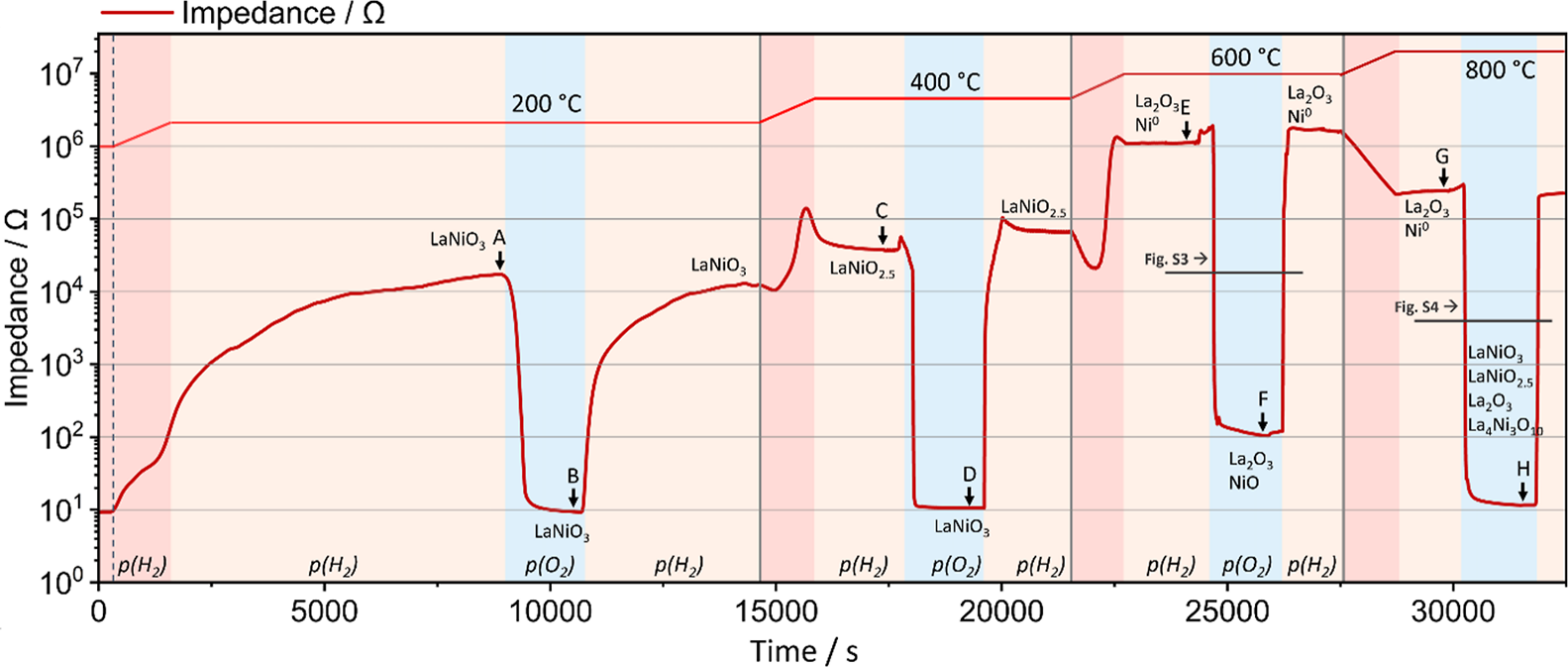
Impedance profile during an equilibration-based reduction–oxidation approach (10% H_2_ or O_2_ in He) between 200 and 800 °C. Dark red indicates heating steps, light red equilibration steps, and blue exposure to oxygen. The top red profile depicts the temperature evolution. The respective holding times at the respective equilibration steps are 5 min in He and 20 min in O_2_ each. For equilibration in H_2_, the times were adjusted to the temperatures: 120 min at 200 °C before the intermittent O_2_ treatment, 60 min after the O_2_ treatment; 30 min at 400 °C before the intermittent O_2_ treatment, 25 min after the O_2_ treatment; 25 min at 600 °C before the intermittent O_2_ treatment, 20 min after the O_2_ treatment; 20 min at 800 °C before the intermittent O_2_ treatment, 20 min after the O_2_ treatment.

**Figure 5:**
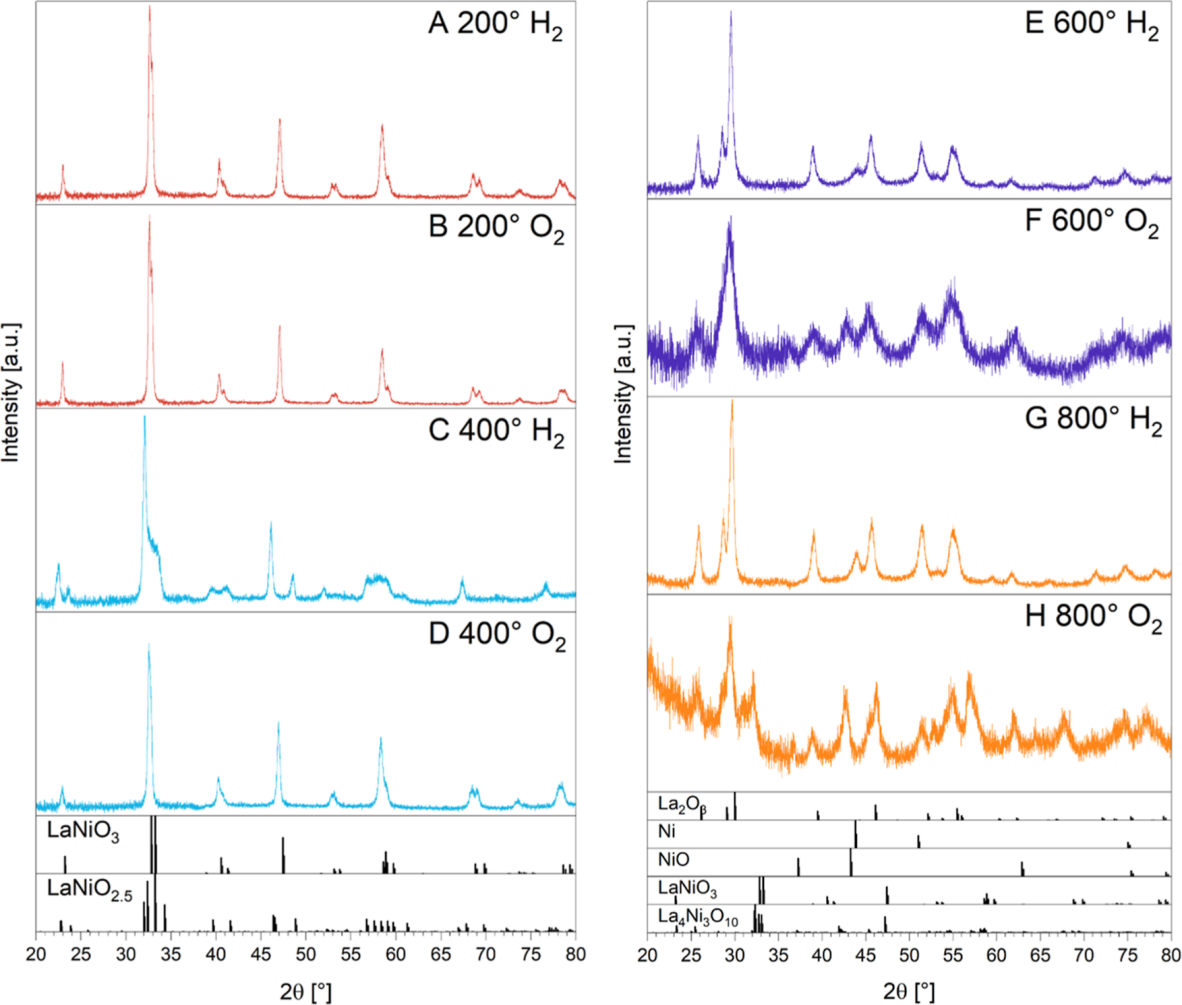
In situ recorded PXRD patterns of LaNiO_3_ between 200 °C and 800 °C in 10% H_2_ and O_2_ in He under ambient conditions, corresponding to selected points A–H in Figure 4. The respective Rietveld refinement to qualitatively pinpoint the present phases is shown in Figure S5.

The decomposition of LaNiO_3_ into its constituent phases Ni + La_2_O_3_ can also be directly seen in electron microscopy images, Figure 6. Ni begins to segregate and agglomerate into distinct nanoparticles, which progressively expand in size at elevated temperatures. At the same time, the distribution of La and O remains homogeneously dispersed throughout the perovskite matrix. At 400 °C, the TEM pictures show an overall uniform Ni distribution; however, very small <10 nm Ni particles agglomerate, predominantly located at the interface between particles. These surface-confined modifications did not affect the parent remaining perovskite structurally, as evidenced by PXRD, or the elemental distribution, as highlighted by EDX mapping. This observation reinforces the concept of the equilibrium-based examination, as the parent perovskite structure remains homogeneous and the redox-induced modifications are predominantly surface-confined. This interpretation aligns with the XPS results, which reveal surface-level changes without perturbation of the bulk lattice. This underlines the structural reversibility upon oxygen exposure and affirms the overall bulk-phase stability of LaNiO_3_/LaNiO_2.5_ during alternating redox cycles.

**Figure 6:**
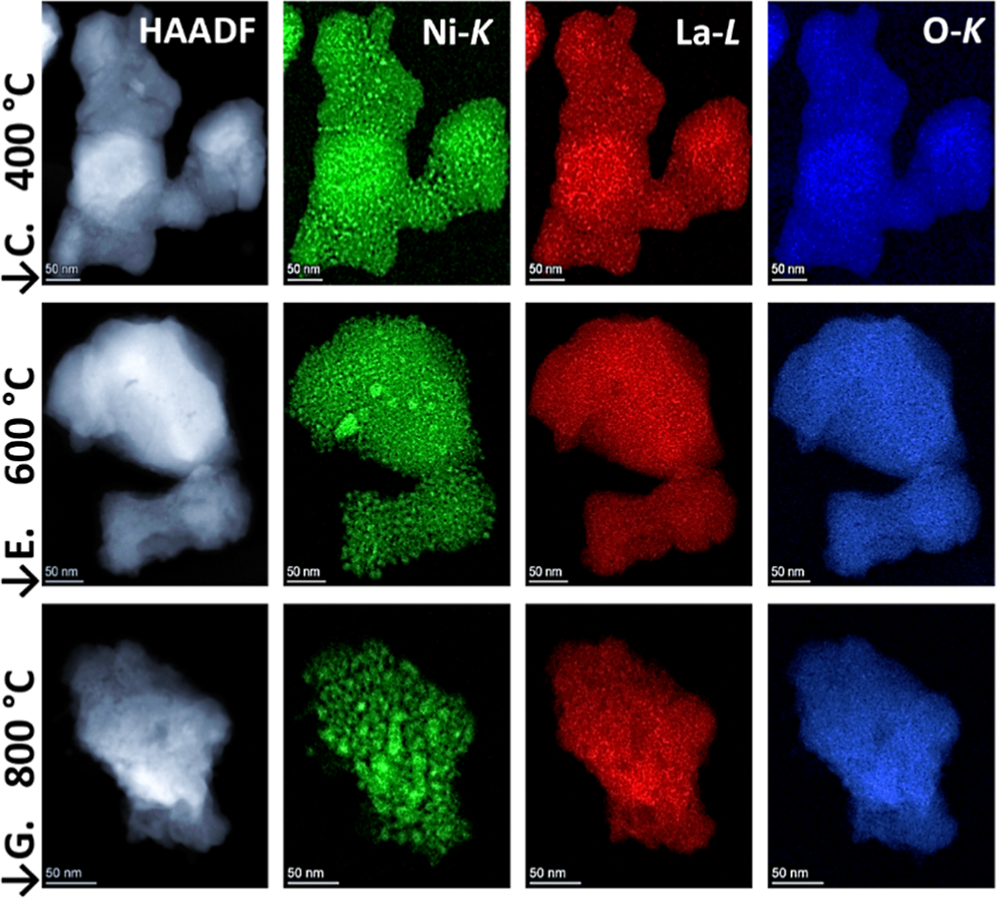
Electron microscopy analysis of LaNiO_3_ after H_2_ equilibration at 400 °C, 600 °C, and 800 °C. Letters C, E, and G indicate the positions in the impedance experiment. HAADF STEM images are shown for each step in the left panel. In addition, chemical analysis by EDX based on the Ni–K (green), La–L (red), and O–K (blue) edges is presented.

### 3.4 Frequency-Dependent Impedance Spectroscopy during Redox Treatment of LaNiO_3_: Elucidating Charging Effects, p-Type Semiconduction, and Inductive Reactance Properties

LaNiO_3_ is intrinsically paramagnetic (i.e., nonmagnetic)^9–11^ and its conduction behavior under reducing and oxidizing atmospheres can be effectively interpreted using defect theory in solids and the associated Brouwer diagrams. In this framework, both the defect concentrations and the resulting electrical conductivity are strongly dependent on oxygen partial pressure *p*(O_2_) and temperature and only a single type of point defect dominates at any particular composition.^37^ If for typical transition metal oxides, where cationic vacancies V_M_^••^ and oxygen vacancies V_O_″ are the dominant defect species, the exact stoichiometric composition V_M_^••^ = V_O_″ is not given, electrons and electron holes help to maintain charge neutrality. Under strongly reducing conditions, such as in H_2_-rich atmospheres, where *p*(O_2_) is extremely low, the thermodynamic driving force favors the release of lattice oxygen, leaving behind oxygen vacancies and electrons.^37,38^ Using Kröger Vink notation (a detailed definition of the formula and associated variables is provided in Supporting Information Section E), this can be formulated as

1 $$O_{O}^{x}⇀0.5O_{2}+V_{O}^{\cdot \cdot }+2e^{-}$$

Consequently, the concentration of electrons and oxygen vacancies rises, while the concentration of cationic vacancies and electron holes decreases (electron holes are annihilated by electrons). However, in the case of LaNiO_3_, we suggest that eq 1 continues by a follow-up reaction, as Ni has multiple possible valence states. After the ad hand reaction, Ni finds itself in the energetically unfavorable +3 state:

Step 1 (eq 1 applied to our example):

2 $$O_{O}^{x}+{Ni}_{Ni}^{\cdot }⇀0.5O_{2}+2e^{-}+{Ni}_{Ni}^{\cdot }+V_{O}^{\cdot \cdot }$$

Ni^•^_Ni_ hereby refers to Ni^3+^, as we assume Ni^2+^ to be the thermodynamic most desirable state, thus consequently termed Ni_Ni_^*x*^.

Step 2:

3 $$2e^{-}+{Ni}_{Ni}^{\cdot }+V_{O}^{\cdot \cdot }⇀e^{-}+{Ni}_{Ni}^{x}+V_{O}^{\cdot \cdot }$$

As Ni favors its +2 state, more oxygen vacancies and fewer electron holes are present, which reduces the number of electronic charge carriers and, thus, the conductivity. On the other hand, when the oxygen partial pressure increases, oxygen from the atmosphere is incorporated into the lattice, oxygen vacancies are compensated, and cationic vacancies and electron holes (h^•^) are formed:

4 $$M_{M}^{x}+0.5O_{2}⇀V_{M}^{\prime \prime }+2h^{\cdot }+MO$$

Charge transport in crystalline media is either electronic by electrons and electron holes or ionic by charged point defects like interstitials and vacancies. The increase in electron hole concentration facilitates electronic charge transport and thus the conductivity of the sample. This also perfectly coincides with our measurement data, where LaNiO_3_ rises in impedance in reducing atmospheres, while it stays a full electronic conductor at 25 °C or when exposed to an oxygen atmosphere.^1,37,38^

To establish impedance spectroscopy as a viable technique for the analysis in a catalytic process reactor environment, frequency-dependent impedance measurements were conducted at 200 °C, 400 °C, 600 °C, and 800 °C under both reducing, H_2_, and oxidizing, O_2_, atmospheres. The measurements were performed in accordance with the selected points A–H in Figure 4, representing key stages in the structural and electronic evolution of the material.

The measurements are displayed as Bode plots, showing the modulus of the impedance and the phase shift as a variation of the perturbation frequency. The full frequency range is always plotted on the left side, and a magnification of the especially changing high-frequency region is always plotted on the right side. The measurements were done for LaNiO_3_, as well as two reference compounds, La_2_O_3_ and NiO. Distinct differences are easily seen and LaNiO_3_ features itself not simply as a superposition of NiO and La_2_O_3_. The data trajectories are labeled according to the subsequent structures, and the surrounding atmosphere is superscripted. The labeling of the impedance modulus is featured left (on the full-scale image), the labeling of the phase angles on the right-hand side of the magnification. Overall, there is a clear tendency for the semiconducting samples that the data are less noisy at high temperatures, while at low temperatures, a higher fluctuation especially at low frequencies (below 1 kHz) becomes evident. La_2_O_3_, typically for a semiconductor, features for all measured temperatures the highest impedance modulus reaching up to 10 *G*Ω at 200 °C. It is hardly influenced by the gas atmosphere, with a slight tendency to be more conductive in an O_2_ atmosphere (800 °C). La_2_O_3_ is not reaching its steady state region below 400 °C, indicating charging and the presence of capacitive effects (panel E, Figure 7).^39^ NiO behaves entirely differently, and its conductivity is highly dependent on the reaction conditions. While insulating at 200 °C under hydrogen, its p-type semiconduction properties are evident in the O_2_ atmosphere, featuring a plateau onset frequency from 8 kHz, from where the modulus impedance remains constant and the semiconduction charge transfer is the conduction determining process.^1,37^

**Figure 7:**
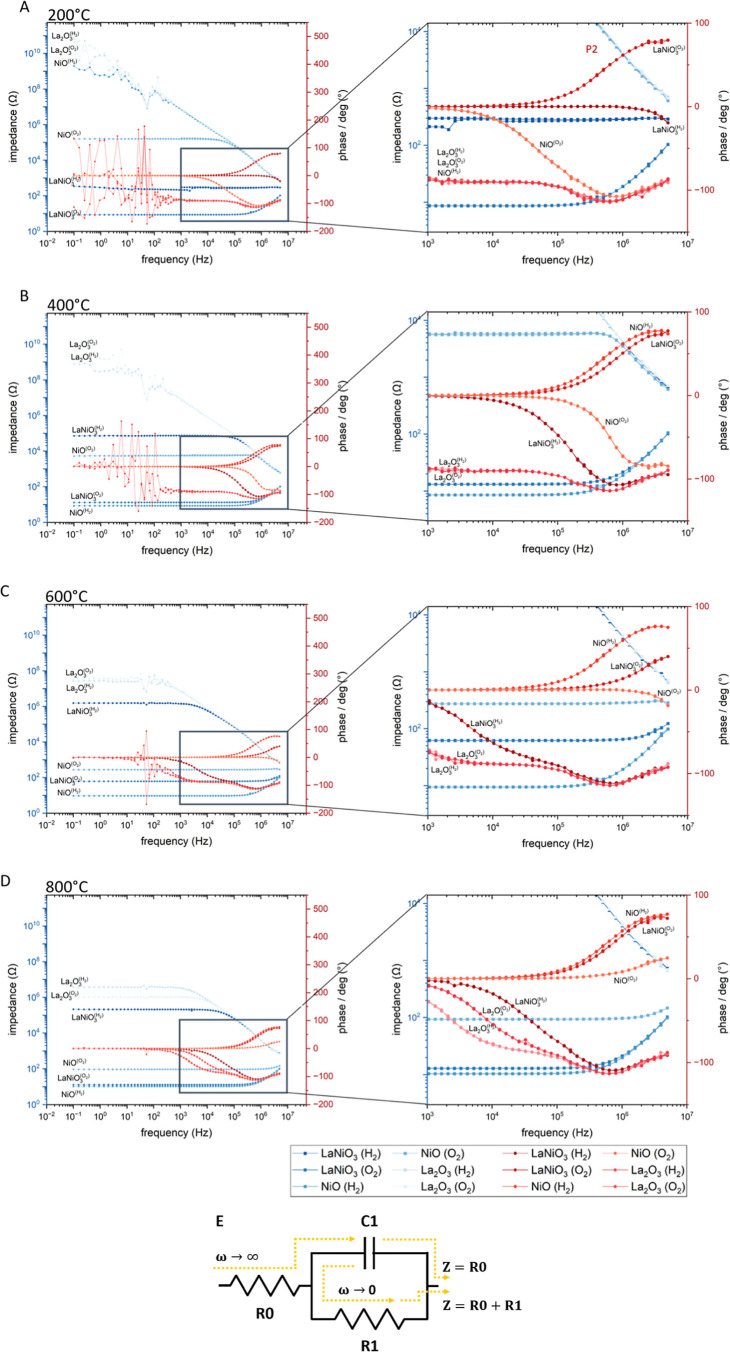
Frequency-dependent impedance measurements displayed as Bode plots at 200 °C, 400 °C, 600 °C, and 800 °C (A–D) with magnification of the high frequency region at the right. (E) shows the utilized Randle’s equivalent circuit, demonstrating the charging effects at low frequencies.

Reaction equations for p-type semiconduction in NiO:

5 $$0.5O_{2}\left(g\right)⇌O^{2-}+V_{{Ni}^{2+}}$$

6 $$V_{{Ni}^{2+}}⇌V_{{Ni}^{2+}}^{-}+h^{\cdot }$$

7 $$V_{{Ni}^{2+}}^{-}⇌V_{{Ni}^{2+}}^{2-}+h^{\cdot }$$

Therefore, the concentration of charge carriers in NiO is dependent on the oxygen partial pressure in the reactor environment, and thus, the conductivity is given as

8 $$\sigma =f\left(P_{O_{2}}^{1/n}\right)$$

LaNiO_3_ remains fully conducting in O_2_, while in a H_2_ atmosphere, it adopts a slightly more resistive behavior (cf. also Figure 3), while still remaining bulk structure-stable LaNiO_3_. Investigating the phase angles, LaNiO_3_ (O_2_) stands out by featuring an inductive effect at high frequencies, which is commonly associated with its metallic conduction and persistent paramagnetism.^40^ We have elaborated on the explanation of inductive features in the Supporting Information in more detail.

In a hydrogen atmosphere, although without bulk structural changes at 200 °C, LaNiO_3_ exhibits a tendency toward a capacitive behavior at very high frequencies of 5 GHz. La_2_O_3_ (H_2_ and O_2_) and NiO feature full capacitive behavior at high frequencies and start exhibiting high fluctuations below 1 kHz. NiO (O_2_) features a full capacitive (>90°) effect at high frequencies but behaves like an Ohmic resistor from 8 kHz on. The time scale of the conduction mechanism therefore lies above this frequency and must be part of the peak feature presented in the phase angle plot, within which also the process frequency for eq 5 must accordingly lie.

At 400 °C, the modulus impedances of La_2_O_3_ (O_2_ and H_2_) behave similar as at 200 °C, however, reaching smaller end and starting impedances, aligning with the data in Figure S1, panel e. The phase angles exhibit the same capacitive behavior as at 200 °C; however, it becomes apparent that the data fluctuation at low frequencies decreases. NiO takes an earlier plateau frequency in the O_2_ atmosphere, which aligns with a temperature-assisted semiconduction process. A dramatic change, however, happens in the H_2_ atmosphere, as is also apparent in Figure S2 panel f because the oxide changes into Ni^0^ at 310 °C, and therefore, the metallic (and ferro/paramagnetic, *T*_Curie_ = 358 °C) Ni adopts the impedance modulus of LaNiO_3_ and the phase angle changes toward the same inductive electronic characteristics. Also, the phase angle moves toward higher frequencies for NiO in O_2_. LaNiO_3_ becomes the oxygen-deficient LaNiO_2.5_ phase and therefore also the impedance modulus as well as the phase angle changes toward a more capacitive nature in H_2_. In O_2_, however, it oxidizes back to LaNiO_3_, which yields the same properties as those at 200 °C.

At 600 °C, La_2_O_3_ features for the first time a plateau frequency at 400 Hz, independent of the atmosphere, less fluctuation in the low frequency region, and behaves like an Ohmic resistor at low frequencies. Two processes become apparent in the high frequency branch at 4 kHz and 70 kHz, which correlate well with typical onset frequencies for bulk (70 kHz) and grain boundary (4 kHz) effects.^41,42^ The frequency-dependent NiO (O_2_) data also corresponds very well to the modulus measurements on the reference structure in Figure S2 panel f. NiO (H_2_) remains reduced in the metallic state, featuring again an inductive property at high frequencies >10^5^ Hz, which apparently stays constant at all temperatures 400 °C, 600 °C, and 800 °C. LaNiO_3_, which decomposes into La_2_O_3_ and Ni^0^, features semiconducting properties of La_2_O_3_ with a plateau frequency at 3 kHz. It also shows two underlying processes in the phase angle around 60 kHz, like La_2_O_3_, but with the second process around 25 kHz at a significantly higher frequency than that of La_2_O_3_ (4 kHz).

At 800 °C, the impedances of the semiconductors become once again smaller. The phase angle shift, which is indicative for the occurring processes, drifts to higher frequencies, showing the thermal effect on conduction (low frequencies exhibit no phase shift anymore; all processes move to higher frequencies and are consequently more easily accessible). The two processes can—in accordance with the current literature^41,42^ —only be ascribed to bulk (75 kHz) and grain boundary (10 kHz) conduction, as they match the typical onset frequencies for these processes. In contrast to 600 °C, LaNiO_3_ (O_2_) becomes purely metallic again, which is due to the back-oxidation into the perovskite structure and the correlating electronic features.^9,10^ NiO (H_2_) remains unchanged. Ni (O_2_) corresponds to the data displayed in Figure S2, panels a–c,f, decreasing in impedance and following the phase shift to moderately higher frequencies. Table 1 recapitulates the key findings upon the frequency-dependent testing of LaNiO_3_ and reference structures.

**Table 1:** Summary of Frequency-Dependent Phase/Impedance Behavior of LaNiO_3_ and Reference Structures La_2_O_3_ and NiO

sample and treatment	chemical constitution/state	phase/impedance behavior
LaNiO_3_ in O_2_	always with Ni^3+^ in surface near region	inductive at all *T* at high frequencies
LaNiO_3_ in H_2_	with Ni^2+^ in surface near region	capacitive at high frequencies
La_2_O_3_ in O_2_ and H_2_	La^3+^ in surface near region and bulk	always capacitive, independent of gas phase and *T*
NiO in O_2_	Ni^2+^ and cationic vacancies V_Ni_^2+^ in surface near region p-type semiconduction	always capacitive, except 800 °C due to sufficiently strong thermal decomposition into Ni^0^ → partially inductive
		
NiO in H_2_	Ni^2+^	inductive once Ni^0^ is formed → only capacitive at 200 °C
	Ni^0^ from 400 °C	

To determine the apparent activation energies of conduction and to take a closer look at the electronic properties of the materials, the impedance data was transferred into conductivity (we refer to the experimental part for calculation), and Arrhenius plots have been established for La_2_O_3_, NiO, and LaNiO_3_ in the employed gaseous environments. The latter are given in the Supporting Information (Figure S6), and the calculated results are presented in Table S1. Figure 8 gives an all-linear plot, in which the metallic and semiconductive properties of LaNiO_3_ are simultaneously shown in one graph. While LaNiO_3_ preserves its metallic conduction properties in O_2_ and He atmospheres over the whole temperature range (cf. Figure S2), it transforms (point A, Figure 1) and further decomposes (points B–G) into its constituents during H_2_ treatment, leading to a purely semiconducting material. Thus, to compare metallic and semiconducting behavior and make this also graphically visible, we decided to split the figure in two. To make semiconductive conduction ( $\sigma =\sigma_{0}\cdot e^{-E_{C}/RT}$ ) appear linear, the according data was transferred into an Arrhenius plot by plotting ln­(σ Ω^–1^ m^–1^) versus 1000/*T*, while simultaneously, the data for metallic conduction (*R* = *R*_0_·(1 + α*T*)) is presented linearly as resistance versus temperature (in °C). This representation allows the reader to more intuitively associate the corresponding conduction process as a linear slope, facilitating and accentuating visual interpretability. To ensure consistency and comparability, the *x*-axes are matched to correlate at 800 °C (= 0.93 1000/*T*/K^–1^) and despite the different mathematical nature of linear and exponential spacing also match at 92 °C. Metallic, as well as semiconduction data, level out in a plateau at this value, representing a stable and finalized conduction state. LaNiO_3_ features a linear slope in He and O_2_ atmospheres in the impedance data with a slightly steeper increment in O_2_ (0.0023 Ω °C^–1^ (*R* ^2^ = 0.999) vs 0.0020 Ω °C^–1^ (*R* ^2^ = 0.999)). In H_2_, however, LaNiO_3_ delivers six steps of different conduction resistances that are not solely attributed to different phases. Starting from the highest temperature, La_2_O_3_ + Ni were prescinded between 0.94 and 1.18 K^–1^, LaNiO_2.5_ + La_2_O_3_ + Ni between 1.21 and 1.24 K^–1^, LaNiO_2.5_: 1.31–1.5 K^–1^, LaNiO_3_ + LaNiO_2.5_: 1.55–1.59 K^–1^, and LaNiO_3_ between two regions: 1.65–2.05 and 2.28–2.9 K^–1^. The latter values stress the presence of two conduction properties in LaNiO_3_, which can be associated with the surface structural changes, presented in the in situ XPS measurements in Figure 3. To elucidate further information about these conduction regions, the apparent activation energies of conduction (plotted in Figure S7) were calculated from the Arrhenius plots (Figure S6) and are summarized in Table S1.

**Figure 8:**
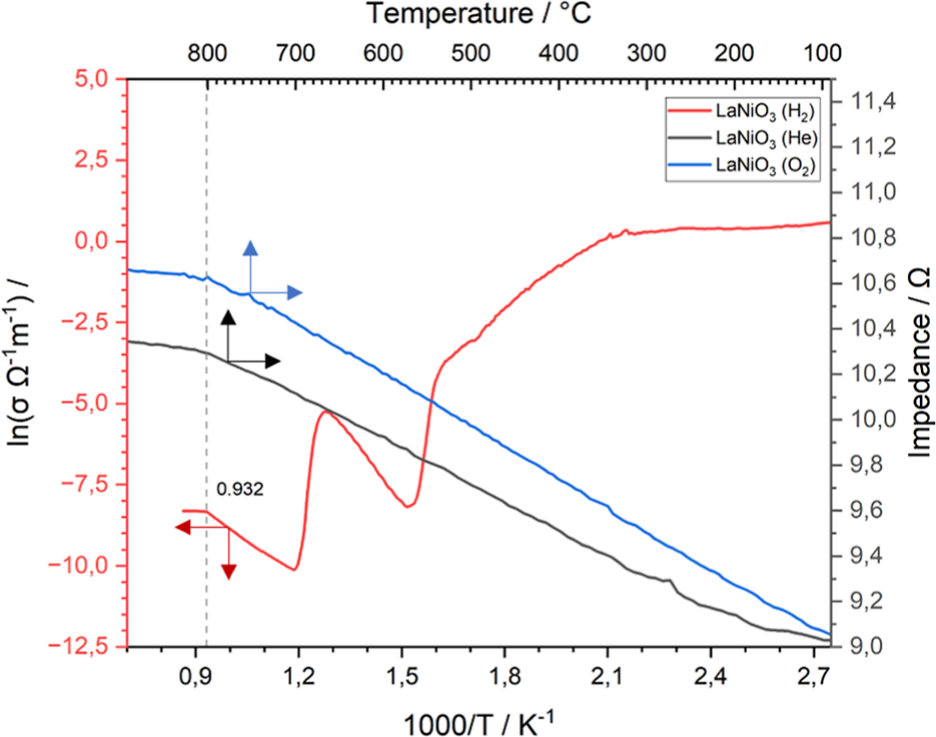
Linearized Arrhenius-type ln­(σ) versus 1000/plot of LaNiO_3_ in H_2_ and impedance of the metallic LaNiO_3_ in He and O_2_ versus the temperature in °C in a linear impedance vs temperature plot.

## 4 Conclusions

Our in situ impedance tracking of LaNiO_3_ under a reduction atmosphere demonstrated to be highly potent for the in situ detection of bulk and surface-bound redox processes, ionic transport processes, and structural phase transitions. It proved simultaneously the possibility to assess the electronic structure of oxygen-deficient phases and the decomposition into multiphase systems, such as La_2_O_3_ and Ni. We have also shown that correlation of impedance and DDTA data allows the quantitative decomposition of LaNiO_3_, giving rise to distinct exothermic and endothermic signals matching the impedance results and surface redox transformations. Equilibrium-focused impedance analysis revealed a pronounced kinetic delay in structural transformations at lower temperatures. Impedance spectroscopy demonstrated exceptional sensitivity to surface reorganization upon redox cycling, including the reoccupation of oxygen vacancies and remodeling of structural defects. This allowed clearly differentiating between reversible and irreversible transformations, such as the irreversible decomposition into La_2_O_3_ and Ni, followed by partial oxidation of metallic Ni into NiO rather than full reconstruction to LaNiO_2.5_. Ultimately, impedance spectroscopy proved the partial reformation of the perovskite phase (LaNiO_3_) at 800 °C, underscoring the method’s effectiveness/efficacy in tracking local and global phase evolution and reversibility. Frequency-dependent investigations and impedance modulus data demonstrate the difficulty to assess low-frequency (<100 Hz) data of La_2_O_3_ and NiO (in H_2_) samples at low temperatures and reveal charging effects that we elaborated upon from a simplified Randle-type equivalent circuit model. In contrast, LaNiO_3_ and NiO (in O_2_) are displayed well at all temperatures. LaNiO_3_ exhibits an inductive reactance in an oxygen atmosphere. Oxygen-deficient LaNiO_2.5_ and the irreversible decomposition into La_2_O_3_ + Ni are also very well visible in the frequency-dependent investigations and expressed via the increasingly capacitive nature of the samples. p-type semiconduction profoundly influences the impedance behavior of NiO under redox conditions and was also found to be the key conduction element of LaNiO_3_ decomposition at 600 °C. This highlights the strength of impedance spectroscopy as a noninvasive, highly responsive marker method for surface chemistry, defect dynamics, and bulk structural transformations during redox experiments in LaNiO_3_.

## Supplementary Material



## Data Availability

Data will be made available on a repository under doi: 10.48323/dgecx-n7j32

## Notes

Section A: Rietveld refinement of the PXRD data of Figure 1; Section B: impedance response of LaNiO_3_, NiO, and La_2_O_3_ upon heating in He, H_2_, and O_2_ atmospheres; Section C: in situ PXRD experiment detailing the phase transition of LaNiO_3_ at 600 °C under equilibrium conditions; Section D: in situ PXRD experiment detailing the phase transition of LaNiO_3_ at 800 °C under equilibrium conditions; Section E: Rietveld refinement of the PXRD data of Figure 5; Section F: enthalpy calculations; Section G: Kröger Vink notation: formalism and associated variables and inductive features; Section H: calculation of activation energies; and Section I: details of the XPS analysis (PDF)

## References

[1] Imelik, B. ; Vedrine, J. C. Catalyst Characterization. In Physical Techniques for solid Materials; Springer Verlag GmbH, 1994.

[2] Bonmassar N., Bekheet M. F., Schlicker L., Gili A., Gurlo A., Doran A., Gao Y., Heggen M., Bernardi J., Klötzer B. (2020). InSitu-Determined Catalytically Active State
of LaNiO_3_ in Methane Dry Reforming. ACS Catal..

[3] Nezhad P. D. K., Bekheet M. F., Bonmassar N., Schlicker L., Gili A., Kamutzki F., Gurlo A., Doran A., Gao Y., Heggen M. (2021). Mechanistic
in situ insights into the formation,
structural and catalytic aspects of the La_2_NiO_4_ intermediate phase in the dry reforming of methane over Ni-based
perovskite catalysts. Appl. Catal., A.

[4] Malleier C., Penner S. (2024). Metal-Perovskite Interfacial Engineering
to Boost Activity
in Heterogenous Catalysis. Surfaces.

[5] Cao P., Tang P., Bekheet M. F., Du H., Yang L., Haug L., Gili A., Bischoff B., Gurlo A., Kunz M., Dunin-Borkowski R. E., Penner S., Heggen M. (2022). Atomic-Scale
Insights into Nickel Exsolution on LaNiO_3_ Catalysts via
In Situ Electron Microscopy. J. Phys. Chem.
C.

[6] Winterstein T. F., Malleier C., Klötzer B., Kahlenberg V., Hejny C., Bekheet M. F., Müller J. T., Gurlo A., Heggen M., Penner S. (2024). Molybdate-based double
perovskite materials in methane dry reforming. Mater. Today Chem..

[7] Neto R. C., Sales H. B. E., Inocêncio C. V.
M., Varga E., Oszko A., Erdohelyi A., Noronha F. B., Mattos L. V. (2018). CO_2_ reforming of methane over supported LaNiO_3_ perovskite-type
oxides. Appl. Catal., B.

[8] Bekheet M. F., Delir Kheyrollahi Nezhad P., Bonmassar N., Schlicker L., Gili A., Praetz S., Gurlo A., Doran A., Gao Y., Heggen M. (2021). Steering
the Methane Dry Reforming Reactivity of Ni/La_2_O_3_ Catalysts by Controlled In Situ Decomposition of Doped La_2_NiO_4_ Precursor Structures. ACS Catal..

[9] Guo H., Li Z. W., Zhao L. (2018). Antiferromagnetic correlations
in the metallic strongly correlated transition metal oxide LaNiO_3_. Nat. Commun..

[10] Liu C., Humbert V. F. C., Bretz-Sullivan T. M. (2020). Observation of an antiferromagnetic
quantum critical point in high-purity LaNiO_3_. Nat. Commun..

[11] Zhang J., Zheng H., Ren Y., Mitchell J. F. (2017). High-Pressure Floating-Zone
Growth of Perovskite Nickelate LaNiO_3_ Single Crystals. Cryst. Growth Des..

[12] Vijatovic M. M., Bobic J. d., Stojanovic B. D. (2008). History and challenges of barium
titanate: part I. Sci. Sinter..

[13] Malyshev S. A., Shlyakhtin O. A., Huang S., Timofeev G. M., Mazo G. N., Roslyakov I. V., Vasiliev A. V., Kustov A. I. (2024). Metal-oxide nanocomposites
by low temperature exsolution from perovskite-like La nickelates:
Synthesis, morphology, and catalytic properties in CO_2_ hydrogenation. Mater. Res. Bull..

[14] Moriga T., Usaka O., Nakabayashi I., Kinouchi T., Kikkawa S. (1995). Kanamaru,
Characterization of oxygen-deficient phases appearingin reduction
of the perovskite-type LaNiO_3_ to La_2_Ni_2_O_5_. Solid State Ionics.

[15] DIFFRAC. TOPAS 7.0; Bruker, 2025.

[16] Haug L. (2023). A laboratory-based multifunctional
near ambient pressure X-ray photoelectron
spectroscopy system for electrochemical, catalytic, and cryogenic
studies. Rev. Sci. Instrum..

[17] García-Muñoz J. L., Rodríguez-Carvajal J., Lacorre P., Torrance J. B. (1992). Neutron-diffraction
study of RNiO_3_ (R = La,Pr,Nd,Sm): Electronically induced
structural changes across the metal-insulator transition. Phys. Rev. B.

[18] Alonso J. A., Martinez-Lope M. J., Garcia-Mufioz J. L., Fernandez M. T. (1997). Crystal
structure and magnetism in the defect perovskite LaNiO_2.5_. Phys. B.

[19] Gokhan
Ünlü C., Burak Kaynar M., Simsek T., Tekgül A., Kalkan B., Özcan S. (2019). Structure and magnetic properties
of (La_1‑x_Fe_x_)­FeO_3_ (x = 0,
0.25, 0.50) perovskite. J. Alloys Compd..

[20] Conway J. O., Prior T. J. (2019). Interstitial nitrides
revisited - A simple synthesis
of M_x_Mo_3_N (M = Fe, Co, Ni). J. Alloys Compd..

[21] Barin, I. Thermochemical Data of Pure Substances; VCH Verlagsgesellschaft mbH, 1989.

[22] Chase M. W., Curnutt J. L., Downey J. R., McDonald R. A., Syverud A. N., Valenzuela E. A. (1982). JANAF Thermochemical
Tables, 1982 Supplement. J. Phys. Chem. Ref.
Data.

[23] Haynes, W. M. ; Lide, D. R. ; Bruno, T. J. CRC Handbook of Chemistry and Physics, 97th ed.; CRC Press, 2016.

[24] Chase, M. W. NIST-JANAF Themochemical Tables, 4th ed.; J. Phys. Chem. Ref. Data, 1998.

[25] Kashchiew, D. Nucleation, Basic Theory with Applications; Butterworth-Heinemann, 2000.

[26] Abraham, F. F. Homogeneous Nucleation Theory; Academic Press, 1974.

[27] Liu X. Y. (2000). Heterogeneous
nucleation or homogeneous nucleation?. J. Chem.
Phys..

[28] Biesinger M. C., Payne B. P., Grosvenor A. P., Lau L. W. M., Gerson A. R., Smart R. S. C., Smart R. St. C. (2011). Resolving surface chemical states
in XPS analysis of first row transition metals, oxides and hydroxides:
Cr, Mn, Fe, Co and Ni. Appl. Surf. Sci..

[29] Biesinger M. C., Payne B. P., Lau L. W. M., Gerson A., Smart R. S. C. (2009). X-ray
photoelectron spectroscopic chemical state quantification of mixed
nickel metal, oxide and hydroxide systems. Surf.
Interface Anal..

[30] Born A., Johansson F. O. L., Leitner T. (2021). Separation of surface
oxide from bulk Ni by selective Ni 3p photoelectron spectroscopy for
chemical analysis in coincidence with Ni M-edge Auger electrons. Sci. Rep..

[31] Moulder, J. F. ; Stickle, W. F. ; Sobol, P. E. ; Bomben, K. D. Handbook of X-ray Photoelectron Spectroscopy; Perkin-Elmer Corporation, 1992.

[32] Norton P. R., Tapping R. L., Goodale J. W. (1977). A Photoemission study of the interaction
of Ni(100), (110) and (111) surfaces with oxygen. Surf. Sci..

[33] Poulain R., Lumbeeck G., Hunka J., Proost J., Savolainen H., Idrissi H., Schryvers D., Gauquelin N., Klein A. (2022). Electronic and Chemical Properties
of Nickel Oxide Thin Films and
the Intrinsic Defects Compensation Mechanism. ACS Appl. Electron. Mater..

[34] Ai L., Fang G., Yuan L., Liu N., Wang M., Li C., Zhang Q., Li J., Zhao X. (2008). Influence of substrate
temperature on electrical and optical properties of p-type semitransparent
conductive nickel oxide thin films deposited by radio frequency sputtering. Appl. Surf. Sci..

[35] Sasaki S., Fujino K., Takeuchi Y. (1979). X-Ray Determination of Electron-Density
Distributions in Oxides, MgO, MnO, CoO, and NiO, and Atomic Scattering
Factors of their Constituent Atoms. Proc. Japan
Acad. Ser. B.

[36] Song J., Ning D., Boukamp B., Bassat J. M., Bouwmeester H. J. M. (2020). Structure,
electrical conductivity and oxygen transport properties of Ruddlesden–Popper
phases Ln_n+1_Ni_n_O_3n+1_ (Ln = La, Pr
and Nd; n = 1, 2 and 3). J. Mater. Chem. A.

[37] Kröger F. A., Vink H. J. (1956). Relations between the Concentrations of Imperfections
in Crystalline Solids. Solid State Phys..

[38] Tilley, R. J. D. Defects in Solids; John Wiley & Sons, 2008.

[39] Lazanas A. C., Prodromidis M. I. (2023). Electrochemical Impedance Spectroscopy–A Tutorial. ACS Meas. Sci. Au.

[40] Griffiths, D. J. Introduction to Electrodynamics; Cambridge University Pr., 2023.

[41] Coskun M., Polat O., Coskun F. M., Durmus Z., ÇAglar M., Turut A. (2018). The electrical modulus and other
dielectric properties by the impedance
spectroscopy of LaCrO_3_ and LaCr_0.90_Ir_0.10_O_3_ perovskites. RSC Adv..

[42] Mabrouki A. E., Messaoudi O., Dhahri A., Azhary A., Mansouri M., Alfhaid L. (2025). Investigation of the Frequency-Dependent Dielectric
Properties of the As-Prepared LaNiO_3_/Co_3_O_4_ Nanocomposites. ACS Omega.

