# Supplementary material for: In Situ Derived Impedance–Structure Correlation during LaNiO3 Decomposition
Source: J Am Chem Soc. 2025 Nov 20;147(48):44515–28. doi: 10.1021/jacs.5c16809 (PMC12679626; doi:10.1021/jacs.5c16809)
Supplement: Supplementary file 1 [file ja5c16809_si_001.pdf]

Supporting Information

In-situ Derived Impedance – Structure Correlation During $LaNiO_3$ Decomposition

Christoph Malleier,[1] Thomas F. Winterstein,[1] Marc Heggen,[2] Volker Kahlenberg,[3] Bernhard Klötzer,[1] Simon Penner[1,*]

[1]*Institute of Physical Chemistry, University of Innsbruck, Innrain 52c, A-6020 Innsbruck, Austria*

[2] *Ernst Ruska-Centre for Microscopy and Spectroscopy with Electrons, Forschungszentrum Jülich GmbH, Leo-Brandt-Str. 1, D-52428 Jülich, Germany*

[3]*Institute of Mineralogy and Petrography, University of Innsbruck, Innrain 52d, A-6020 Innsbruck, Austria*

*Corresponding author: S. Penner, simon.penner@uibk.ac.at, +4351250758003

Keywords: in situ; impedance; X-ray diffraction; XPS; DDTA; TEM; single perovskite; $LaNiO_3$

Section A Rietveld refinement of the PXRD Data of Figure 1

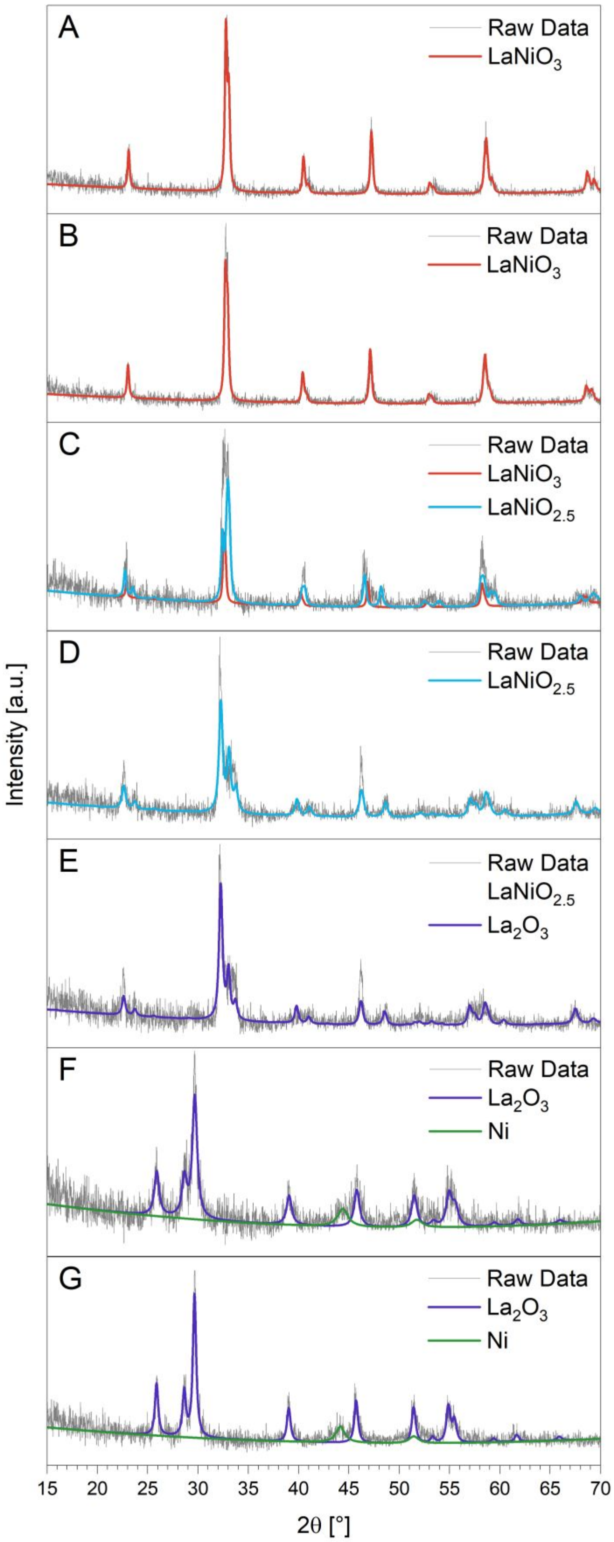

Figure S1: Rietveld refinement of the PXRD data of Figure 1

Section B: Impedance response of $LaNiO_3$, NiO and La_2O_3 upon heating in He, H_2 and O_2 atmospheres

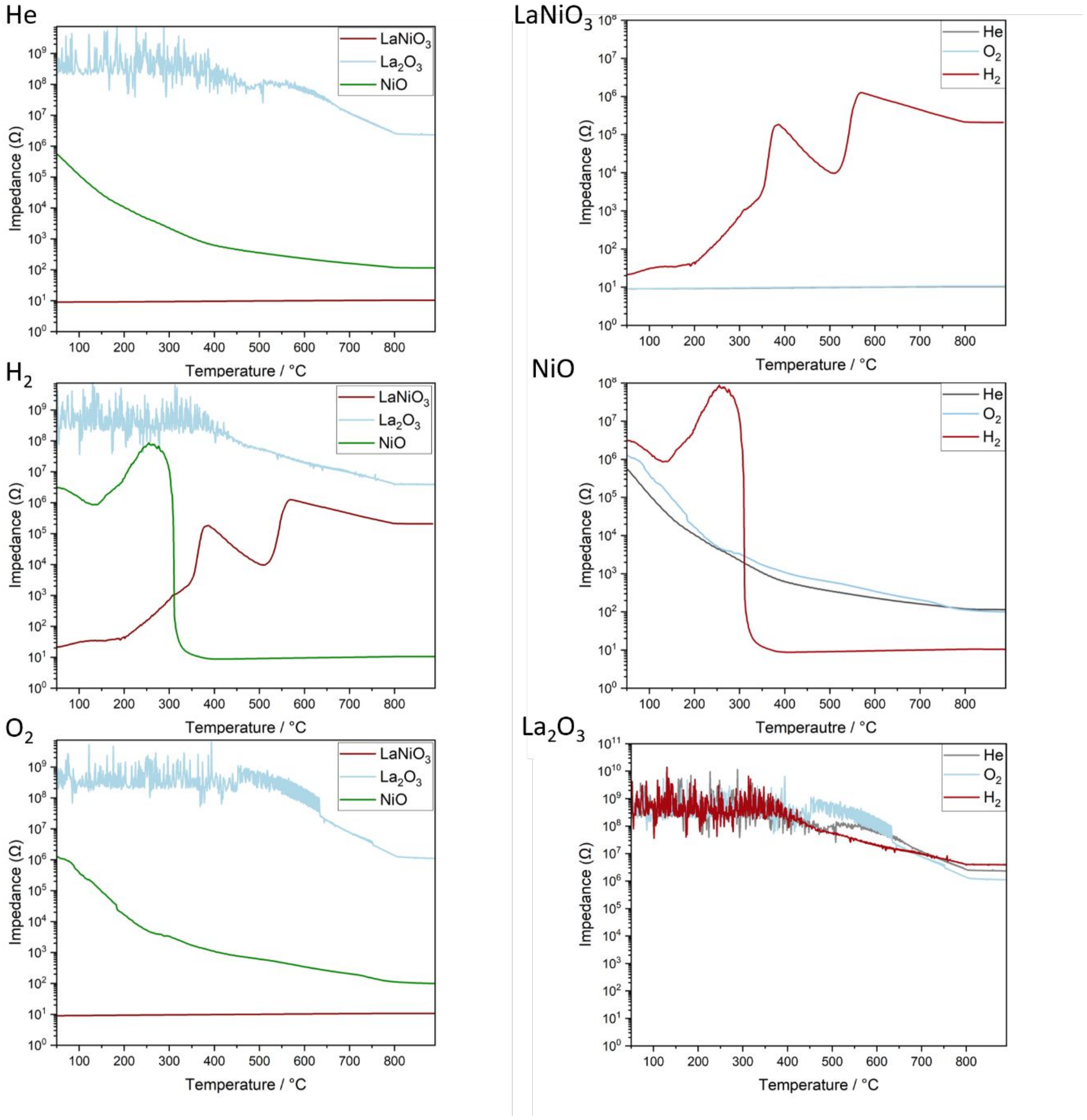

Figure S2: Impedance response of $LaNiO_3$, NiO and La_2O_3 upon heating in He, H_2 and O_2 atmospheres. Experimental conditions as outlined in the main manuscript.

In light of the comprehensive experimental data, the contrast of $LaNiO_3$ with its structurally related materials is important. Figure S1 summarizingly discusses the temperature-dependent impedance profiles of $LaNiO_3$, La_2O_3 and NiO under the different gaseous environments (He, O_2 and H_2). $LaNiO_3$ exhibits metallic conductivity at ambient temperature

across all tested atmospheres, maintaining this behaviour throughout the entire temperature range in both He and O_2 atmospheres. The impedance in these environments increases marginally with temperature, consistent with the well-documented thermal behaviour of metallic conductors ($R = R_0 \cdot (1 + \alpha T)$).[1] In contrast, under H_2 atmosphere the impedance starts to rise – even at room temperature. Given that XRD data (Figures 1 and 5) confirm the absence of any bulk structural transformations at this stage, and incorporating the XPS results (Figure 3) the observed changes result from surface-level alterations. At elevated temperatures, the impedance increases progressively until 280 °C, at which point the decomposition of $LaNiO_3$ initiates and the discussion of Figure 1 in the main paper follows.

The reference structures exhibit distinctly different impedance-temperature behaviour. NiO, a well-established p-type semi-conductor, displays a steady decrease in impedance during heating from 50 °C to 800 °C in O_2 and He atmosphere from 1.3 MΩ (O_2) to 100 Ω and 560 kΩ (He) to 111 Ω with no inflection points, trend reversal or steady state phenomena. In contrast, under H_2 atmosphere, NiO shows a significant reduction profile: The impedance initiates with 3MΩ (50 °C), to decrease to 850 kΩ at 130 °C, until it exhibits a peak shape reaching a maximum of 83 MΩ at 260 °C into the reduction towards metallic Ni at 390 °C (9 Ω). From there on the impedance rises linearly with the temperature until 10 Ω, at 800 °C, as before typical for metallic conductors. Notably, by 320 °C the impedance has already dropped below 50 Ω, suggesting that the major phase transition is nearly complete at this point.

La_2O_3 retains its semiconductive nature across all atmospheres and demonstrates resistance to reduction in H_2. Despite the absence of apparent chemical transformations, the reactor environment does have a significant influence on the impedance data. All measurements start near the detection limit of the instrument in the GΩ scale, 530 MΩ – 1 GΩ (50 °C) but converge to different end impedances at 800 °C: 4.0 MΩ (H_2), 2.4 MΩ (He), 1.2 MΩ (O_2). The profiles until 800 °C run distinctively different. While the H_2 measurement starts its steady

decline at 410°C, the He measurement features a shoulder at 560 °C and falls only after 730 °C below the H_2 data. Entirely different progresses the impedance in O_2 atmosphere where a shoulder evolves at 470 °C and has a significant property change and a sharp bend at 634 °C from where on it sinks towards the lowest impedance of the three gases reaching 800 °C.

Section C In-situ PXRD experiment detailing the phase transition of $LaNiO_3$ at 600 °C under equilibrium conditions

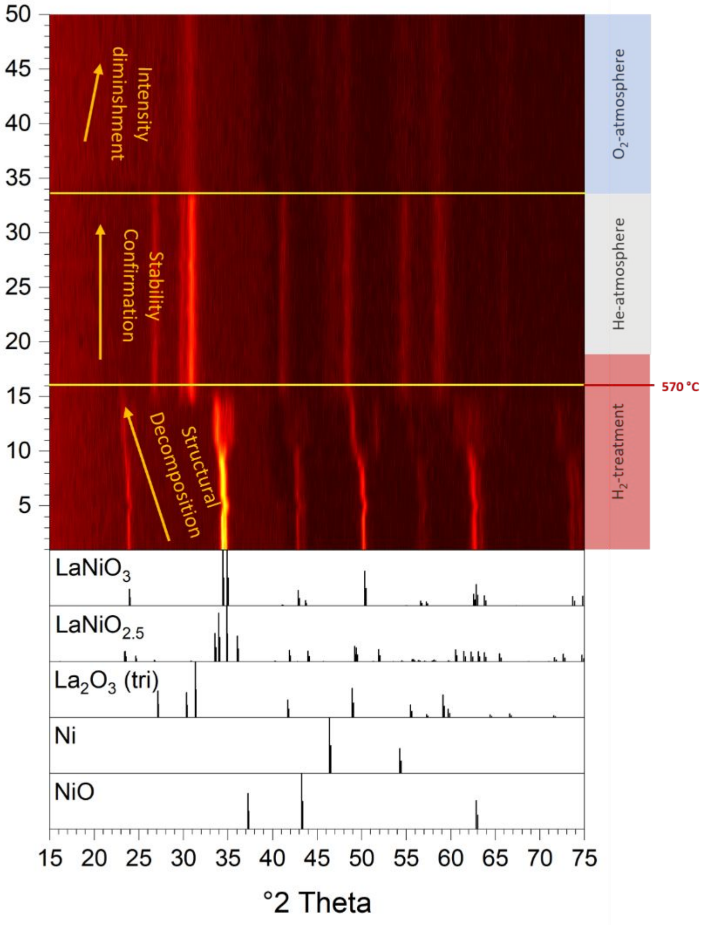

Figure S3: In-situ PXRD experiment detailing the phase transition of $LaNiO_3$ at 600 °C under equilibrium conditions, concordant to point E → F in Figure 4 in the main manuscript.

Section D In-situ PXRD experiment detailing the phase transition of $LaNiO_3$ at 800 °C under equilibrium conditions

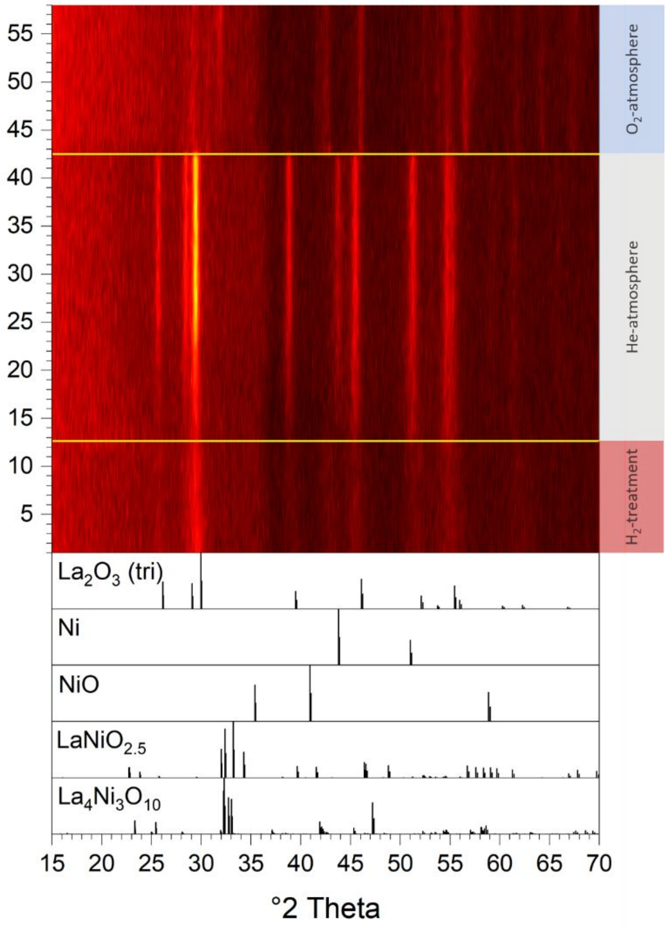

Figure S4: In-situ PXRD experiment detailing the phase transition of $LaNiO_3$ at 800 °C under equilibrium conditions, concordant to point G → H in Figure 4 in the main manuscript.

Section E Rietveld refinement of the PXRD Data of Figure 5

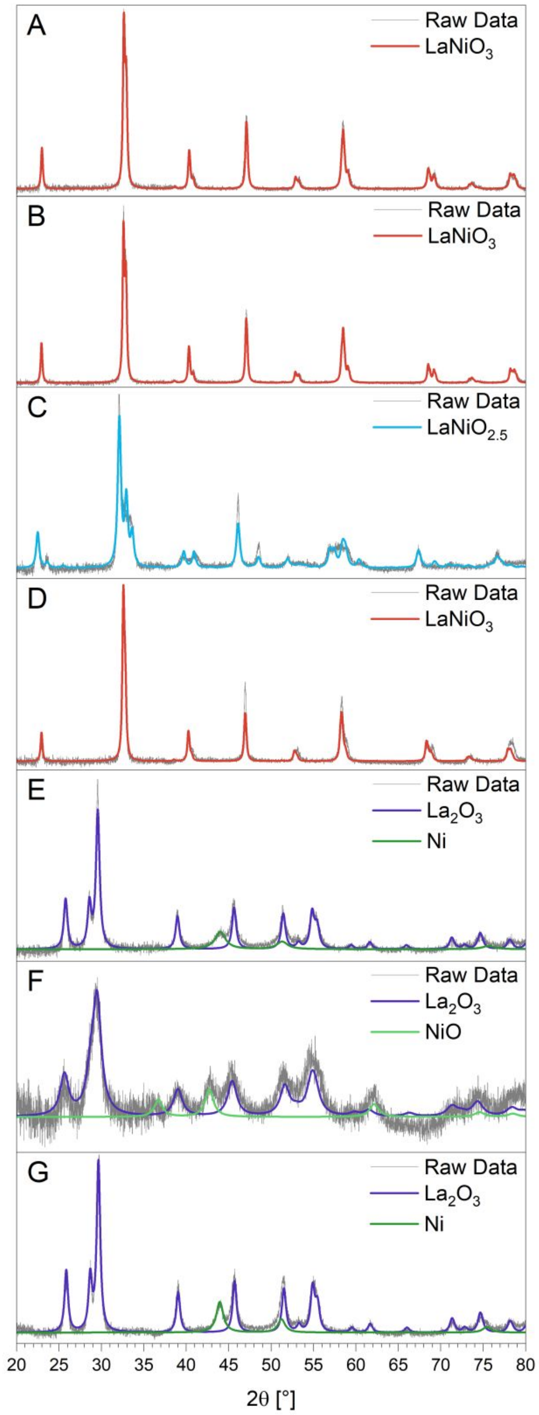

Figure S5: Rietveld refinement of the PXRD data of Figure 5

Section F Enthalpy calculations

Elaborating the enthalpy change from Ni^{3+} to Ni^{2+} through the standard enthalpy of formation for the solid-state reaction[2-5]

$$Ni^{3+}_{solid} \rightarrow Ni^{2+}_{solid} + e^{-},$$

exemplified by the oxides Ni_2O_3 and NiO

$$Ni_2O_3 \rightarrow 2NiO + 0.5O_2,$$

we can use the standard enthalpies of formation to derive the enthalpy change:

$$\Delta H^0_{298}(Ni_2O_3(s)) = -489.5\ kJ/mol$$

$$\Delta H^0_{298}(NiO\ (s)) = -239.7\ kJ/mol$$

$$\Delta H^0_{298}(O_2(g)) = 0\ kJ/mol$$

$$\Delta H^0_{298} = 2NiO + 0.5O_2 - Ni_2O_3 = (2 \cdot (-239.7) + 0) - (-489.5) = +10.1\ kJ/mol$$

The reaction converts 2 Ni^{3+} into 2 Ni^{2+}, releasing O_2. So, per conversion $Ni^{3+} \rightarrow Ni^{2+}$ we obtain:

$$\Delta H^0_{298} = \frac{10.1\ kJ/mol}{2} = \mathbf{5.05\ kJ/mol}$$

In consequence, in a solid Ni_2O_3 lattice, reducing Ni^{3+} to Ni^{2+} is mildly endothermic (~ +5kJ/mol) at standard conditions.

However, most materials overwhelmingly favour the presence Ni^{2+}. What seems like a contradiction at first will be elaborated upon here.

In short, Ni^{2+} is more prevalent because of kinetic stability, electronic configuration, environmental conditions and crystal field stabilization.

Electronic configuration and stability

- Ni^{2+}: [Ar] $3d^8$ → this configuration is relatively stable, especially in octahedral fields as commonly encountered in oxides and salts (e.g., NiO, $Ni(OH)_2$).
- Ni^{3+}: [Ar] $3d^7$ → more oxidized, less stable due to higher effective nuclear charge and tendency to be reduced. The electron deficiency in Ni^{3+} makes it a stronger oxidizing agent and more likely to gain an electron (i.e. to be reduced to Ni^{2+}).

Therefore, although Ni^{3+} can exist, it is more oxidizing and less stable in water or under atmospheric environments (moisture) and typically needs special ligands or structures to be stabilized (e.g. perovskites). Ni^{3+} is typically stabilized in highly oxidizing environments, strong crystal fields (like perovskites) and ligand stabilized complexes like $[NiF_6]^{3-}$. This is why Ni^{3+} is commonly only seen in perovskites such as $LaNiO_3$, but not simple binary oxides.[6]

Thermodynamic instability

Ni^{3+} compounds like Ni_2O_3 decompose at modest temperatures (~600 °C) releasing oxygen and are reduced to Ni^{2+} as NiO.[7]

Crystal field stabilization

In octahedral fields (like NiO), Ni^{2+} ($3d^8$) has a low crystal field stabilization energy. Ni^{3+} ($3d^7$) can be stabilized only in very strong fields (e.g. perovskites like $LaNiO_3$, $PrNiO_3$ or fluorides like K_3NiF_6). Without such stabilization, the lattice or solvent energy is insufficient to hold Ni^{3+} and Ni^{3+} is reduced.[8]

Reasons for discrepancy

Ni_2O_3 is described as a metastable phase.[7] Experimental measurements might capture enthalpy values which include energy released from relaxing structural relaxation and structural defects, which are not reflected in tabulated ΔH_f^0 values derived from ideal crystalline forms. Standard

ΔH_f^0 values result from high-purity phases that do not include defects, surface energy (oxygen vacancies, mixed valence states) and partial decomposition, that are often encompassed in real environmental surroundings.

Standard electrode potential

The standard reduction potentials show this trend also clearly:

$$Ni^{3+} + e^{-} \longrightarrow Ni^{2+} \quad E^0 = 0.48\,V$$

$$Ni^{2+} + 2e^{-} \longrightarrow Ni^0\ (s) \quad E^0 = -0.25\,V$$

The more positive the reduction potential, the stronger the tendency to gain electrons. Thus,

$Ni^{3+} \rightarrow Ni^{2+}$ is favourable (positive E^0, spontaneous)

$Ni^{2+} \rightarrow Ni^0$ is not favourable (negative E^0, non-spontaneous under standard conditions)

From the standard electrode potential and using the Gibbs free energy equation, the reaction enthalpy for $Ni^{3+} \rightarrow Ni^{2+}$.

$$\Delta G^0 = -nFE^0$$

ΔG^0… Gibbs Free Energy / kJ mol^{-1}

n… number of transferred electrons

F… Faraday constant / 96485 C mol^{-1}

$$\Delta G^0 = -1 \cdot 96485\ C/mol \cdot 0.48\ V = -46.312\ kJ/mol$$

With standard entropy values for Ni(l) we can estimate the enthalpy[5]:

$$\Delta G^0 = \Delta H^0 - T\Delta S^0$$

$$\Delta H^0 = \Delta G^0 + T\Delta S^0$$

$$\Delta S^0 \approx 41.49\ J\ mol^{-1}\ K^{-1}$$

$$\Delta H^0 = -46.3\ kJ\ mol^{-1} + 298\ K \cdot 0.04149\ kJ\ mol^{-1}\ K^{-1} = \mathbf{-33.94\ kJ\ mol^{-1}}$$

Section G: Kröger Vink notation: formalism and associated variables. Inductive Features

M_S^C … Kröger Vink notation

M… species that occupies the lattice site /

ion = element symbol, vacant site = V

C… charge of M relative to host lattice /

rel. positive ($\cdot$), neutral (x), negative ($'$) charge

S… lattice site, occupied by M /

ion = element symbol, interstitial = i

To explain the appearance of inductive features in Figure 7 (main paper), Faraday's law of electromagnetic induction expresses the inductive reactance formula for an AC circuit:

$$X_L = 2\pi f L \qquad \text{Eq. S1}$$

X_L… inductive reactance / Ω

f… frequency / Hz

L… inductance / H

At high frequencies, conductors do not behave like ideal wires anymore. Instead, they exhibit inductance due to the changing magnetic fields they create, which in turn oppose the current flow, the inductive effect.[9] When alternating current (AC) flows through a conductor, it creates a time-varying magnetic field around it. The inductive reactance formula, a derivative of Faraday's first law of inductance, Eq. S1 describes this: the changing magnetic field induces a voltage (or electromotive force, EMF) that opposes the change in current with the effect: At high frequencies (rapid current changes), this self-induced EMF becomes significant and resists current flow. Additionally, the resistance of the system increases too, with rising frequency,

due to the so-called skin effect. At high frequencies, current tends to flow only on the surface of the conductor (not throughout its cross-section). This effectively reduces the conducting area and increases AC resistance. The skin effect, again, also reinforces the inductive nature of the conductor, as it enhances the non-uniform distribution of current.[10,11]

Section H: Calculation of activation energies

Table S1: Calculated activation energies in kJ mol^{-1} and standard errors, assigned to the regions in the Arrhenius plots and labelling of the process properties.

	region 1000/T / K^{-1}	*E_A kJ mol^{-1}*	*Err. kJ mol^{-1}*	*property*
$LaNiO_3$	0.94 - 1.18	-59.3	0.2	$H_2^{(1)}$
	1.21 - 1.24	882.6	13.7	$H_2^{(2)}$
	1.31 - 1.5	-116.9	0.5	reaction
	1.55 - 1.59	632.7	6.9	$LaNiO_{2.5}$
	1.65 - 2.05	72.4	0.7	reaction
	2.28 - 2.9	4.6	0.2	La_2O_3 + Ni
La_2O_3	0.94 - 1.1	-133.0	0.8	He
	0.94 - 1.1	-139.0	1.1	$O_2^{(1)}$
	1.103 - 1.218	-109.2	15.2	$O_2^{(2)}$
	0.94 - 1.1	-63.5	1.0	H_2
NiO	0.95 - 2.75	-32.9	0.1	He
	0.92 - 2.525	-35.7	0.2	O_2
	0.95 - 1.5	2.7	0.0	H_2
	1.71 - 1.735	-3287.3	836.6	H_2 pre-activation
	1.925 - 2.12	104.3	1.1	H_2 activation
	2.155 - 2.38	49.4	0.6	H_2 reduction
	2.53 - 2.95	-23.0	0.3	metallic Ni

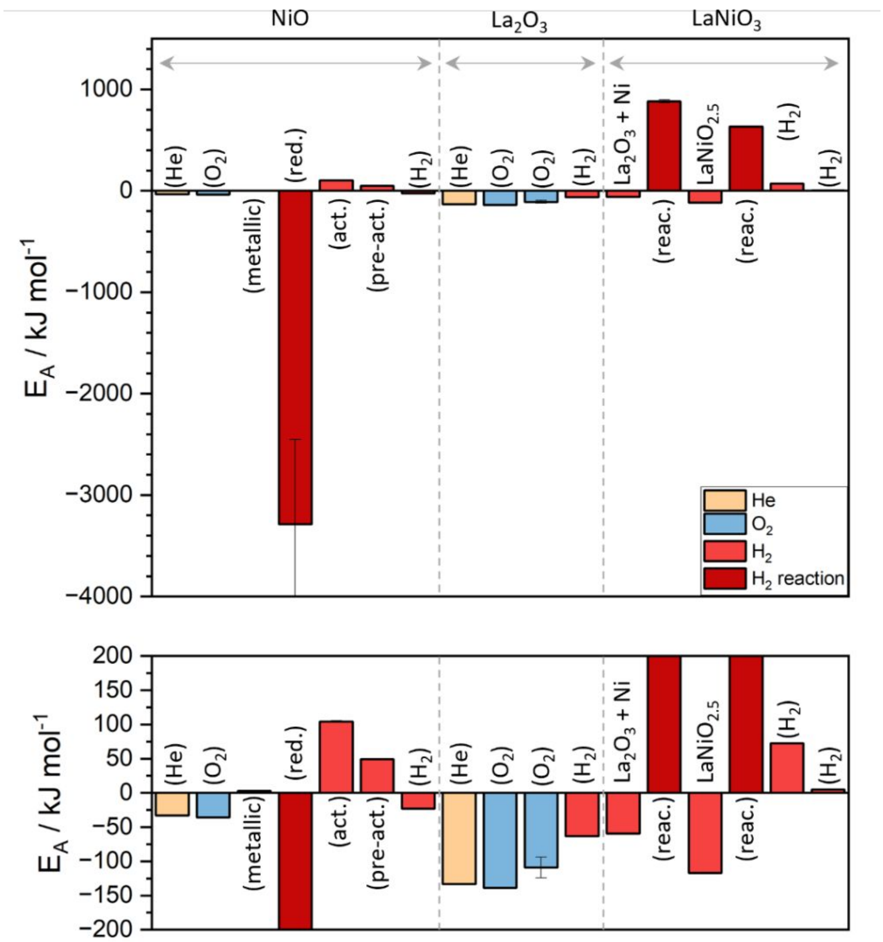

Figure S6: Calculated activation energies of $LaNiO_3$ and reference structures represented as bar-graphs.

Figure S6 compares the apparent activation energies of conduction (with error bars) on $LaNiO_3$, La_2O_3 and NiO in He (light yellow), O_2 (blue) and H_2 (red) atmospheres with a magnification on the bottom. As three processes (indicated as reduction and reactions), attributed to the phase transitions and contain chemical reactions, distinctively outstand the rest of the results, these have been marked in dark red and are discussed separately. Comparing the calculated activations energies distinct differences arise, depending on the gas phase environment. He and O_2 atmospheres feature only negative activations energies of conduction on NiO and La_2O_3, whereby the activation energy of conduction on La_2O_3 is distinctly more negative (4 times in He, 3-4 times in O_2(1 and 2)) than on NiO, underlining the benefits of p-type semi-conduction on NiO. The hydrogen data deliver more processes to distinguish, which

have therefore been separated into four parts. (i) The response of NiO without any reaction to H_2 at rising temperatures. (ii) A pre-activation step where the property of NiO distinctively changes, however, the phase transition is not reached. (iii) Activation, where the reduction in H_2 becomes prepared (clearly different activation energy 49.4 kJ mol^{-1} compared to step (iii) 104.3 kJ mol^{-1}). (iv) The reduction process, which is composed of the conduction through the material and superimposed by the reaction enthalpy and reaction process. La_2O_3 exhibits also a negative H_2 dependency similar to NiO in its first phase, which can be deduced to be a material characteristic in cases without reaction, however with almost 3 times the energy demand (-63.5 comp. -23.0 kJ mol^{-1}) for conduction. As all activation energies on $LaNiO_3$ in H_2 are positive it stands to reason from the NiO and La_2O_3 data that these processes are not attributed to the H_2-dependent response of $LaNiO_3$ to the temperature increase, but to an intercalation of H_2 or surface reaction of H_2 on $LaNiO_3$. The transformation steps into different phases are always significantly higher than sole-phase activation energies, suggesting that these processes are not solely determined by conduction but also by the reactions taking place themselves. The sole phases of $LaNiO_{2.5}$ and La_2O_3 + Ni are semi-conductive again with negative apparent activation energies, where $LaNiO_{2.5}$ is stronger influenced by the temperature than the phase La_2O_3 + Ni. In terms of the reduction process the transformation into $LaNiO_{2.5}$ sets (exothermal process, view Fig. 2) less energy free than the transformation into La_2O_3 + Ni.

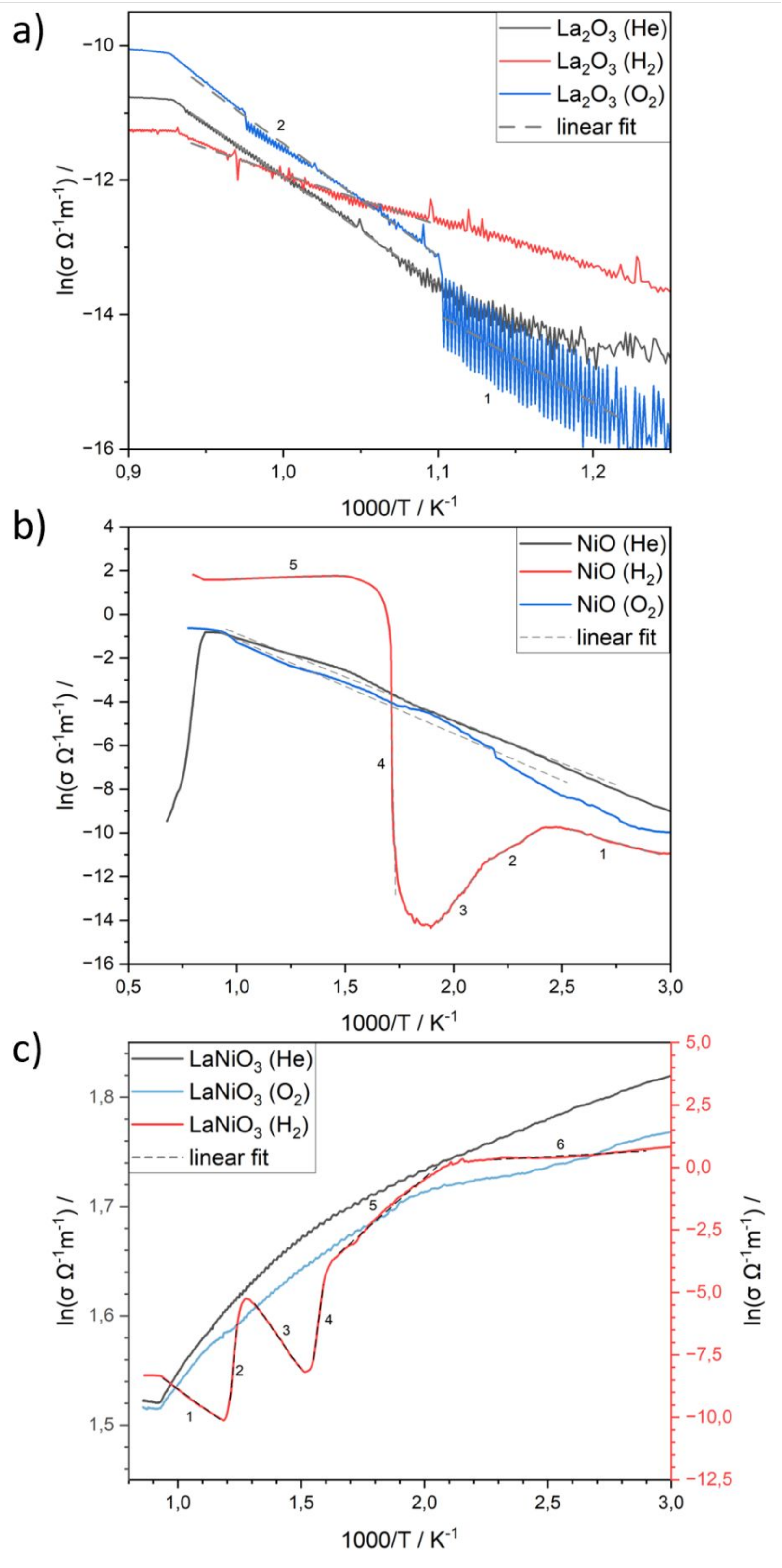

Figure S7: Arrhenius representation of the conductivity data for calculating the activation energy of conduction of a) La_2O_3, b) NiO and c) $LaNiO_3$ in He, O_2 and H_2 atmospheres.

Section I: Details of the XPS analysis

Table S2: Full list of binding energies and properties of the fitted high-resolution X-ray photoelectron spectra of La 3d/ Ni 2p, O 1s and Ni 2s/ La 4d/ Ni 3p regions.

		Component	Pos. / eV BE	FWHM	Area / %
Room Temperature	La 3d + Ni 2p	La 3d 5/2 I	834.58	3.00	22.67
		La 3d 5/2 II	838.36	3.00	21.82
		La 3d 3/2 I	851.24	3.50	21.88
		La 3d 3/2 II	855.98	2.50	12.93
		La MNN Auger	866.12	4.00	4.78
		Ni 2p 3/2	854.57	2.13	8.75
		Ni 2p 3/2 sat.	862.59	3.13	3.65
		Ni 2p 1/2	873.00	3.13	3.53
	O 1s	O 1s lattice	529.58	1.38	37.65
		O 1s vacancy	531.10	1.97	7.70
		O 1s surface	532.66	2.35	54.65
	Ni 3s La 4d Ni 3p	Ni 3p	68.47	4.03	16.73
		Ni 3s	107.83	3.58	13.86
		La 4d 5/2	102.25	2.34	29.58
		La 4d 3/2	105.28	2.86	39.83

		Component	Pos. / eV BE	FWHM	Area / %
100 °C	La 3d + Ni 2p	La 3d 5/2 I	834.54	3.00	22.21
		La 3d 5/2 II	838.32	3.00	22.05
		La 3d 3/2 I	851.24	3.50	21.69
		La 3d 3/2 II	856.02	2.50	12.79
		La MNN Auger	866.19	4.00	4.82
		Ni 2p 3/2	854.51	2.13	8.77
		Ni 2p 3/2 sat.	862.65	3.13	3.79
		Ni 2p 1/2	872.92	3.13	3.87
	O 1s	O 1s lattice	529.64	1.48	44.65
		O 1s vacancy	531.44	1.97	8.94
		O 1s surface	532.87	2.15	46.42
	Ni 3s La 4d Ni 3p	Ni 3p	68.41	4.03	16.56
		Ni 3s	107.66	3.58	16.15
		La 4d 5/2	102.27	2.34	29.30
		La 4d 3/2	105.22	2.66	38.00

		Component	Pos. / eV BE	FWHM	Area / %
28	La 3d	La 3d 5/2 I	834.06	2.65	21.62

		La 3d 5/2 II	838.17	2.68	18.48
		La 3d 3/2 I	850.65	1.94	12.58
		La 3d 3/2 II	855.37	1.91	10.16
		La MNN Auger	864.10	7.81	13.92
		Ni 2p 3/2	852.65	2.13	16.28
		Ni 2p 3/2 sat.	857.76	2.75	2.43
		Ni 2p 1/2	869.79	2.57	4.53
	O 1s	O 1s lattice	529.58	1.48	73.77
		O 1s vacancy	531.43	1.97	16.68
		O 1s surface	532.84	1.85	9.55
	Ni 3s La 4d Ni 3p	Ni 3p	66.66	3.31	16.00
		Ni 3s	108.70	3.78	10.91
		La 4d 5/2	101.92	2.34	34.73
		La 4d 3/2	105.17	2.66	38.35

		**Component**	**Pos. / eV BE**	**FWHM**	**Area / %**
350 °C	La 3d + Ni 2p	La 3d 5/2 I	833.87	2.35	20.37
		La 3d 5/2 II	838.34	2.38	16.38
		La 3d 3/2 I	850.51	1.94	13.42
		La 3d 3/2 II	855.33	1.91	9.39
		La MNN Auger	864.03	7.81	13.73
		Ni 2p 3/2	852.61	1.95	17.32
		Ni 2p 3/2 sat.	857.72	2.75	2.76
		Ni 2p 1/2	869.71	2.57	6.62
	O 1s	O 1s lattice	529.46	1.48	80.82
		O 1s vacancy	531.21	1.97	19.16
	Ni 3s La 4d Ni 3p	Ni 3p	66.43	3.15	16.71
		Ni 3s	109.20	3.78	9.75
		La 4d 5/2	101.98	2.34	34.72
		La 4d 3/2	105.30	2.66	38.82

		**Component**	**Pos. / eV BE**	**FWHM**	**Area / %**
400 °C	La 3d + Ni 2p	La 3d 5/2 I	833.96	2.35	20.41
		La 3d 5/2 II	838.49	2.38	16.16
		La 3d 3/2 I	850.65	1.94	13.26
		La 3d 3/2 II	855.39	1.91	9.42
		La MNN Auger	864.30	7.81	13.05
		Ni 2p 3/2	852.61	1.95	18.17
		Ni 2p 3/2 sat.	857.89	2.75	2.86
		Ni 2p 1/2	869.66	2.57	6.67
	O 1	O 1s lattice	529.58	1.48	79.88

	O 1s vacancy	531.21	1.67	20.10
Ni 3s La 4d Ni 3p	Ni 3p	66.45	3.11	15.71
	Ni 3s	109.24	3.78	10.76
	La 4d 5/2	102.11	2.24	35.19
	La 4d 3/2	105.40	2.56	38.35

References

[1] Imelik, B.; Vedrine, J. C.; *Catalyst Characterization, Physical Techniques for solid Materials*; Springer Verlag GmbH, 1994.

[2] Haynes, W. M.; Lide, D. R.; Bruno, T. J., *CRC Handbook Of Chemistry And Physics*, 97th Edition, CRC Press, 2016.

[3] Chase, M. W., Jr.; Curnutt, J. L.; Downey, J. R., Jr.; McDonald, R. A.; Syverud, A. N.; Valenzuela, E. A., JANAF Thermochemical Tables, 1982 Supplement, *J. Phys. Chem. Ref. Data* **1982**, 11, 695–940. DOI: 10.1063/1.555666

[4] Barin, I.; *Thermochemical data of pure substances*; VCH Verlagsgesellschaft mbH, 1989.

[5] Chase, M.W.; *NIST-JANAF Themochemical Tables*, Fourth Edition, J. Phys. Chem. Ref. Data 1998.

[6] Macintyre, J. E.; Daniel, F.M.; Stirling, V.M.; *Dictionary of Inorganic Compounds*, Springer New York, 1992.

[7] IFA, *Nickel(III)-oxid*, GESTIS-Stoffdatenbank, 2025.

[8] Penn State University, *Introduction to Inorganic Chemistry*, Wikibook, Open Education Resource LibreTexts Project, 2025.

[9] Griffiths, D. J.; *Introduction to Electrodynamics*; Cambridge University Pr., 2023.

[10] Lamb, H.; XIII. On electrical motions in a spherical conductor; *Philosophical Transactions of the Royal Society of London* **1883**, 174: 519–549. DOI: 10.1098/rstl.1883.0013

[11] Kraus, J. D.; *Electromagnetics*, McGraw-Hill Inc., 1992.
